# Influence of Microbiota on Tumor Immunotherapy

**DOI:** 10.7150/ijbs.91771

**Published:** 2024-03-31

**Authors:** Xin Yu, Wenge Li, Zhi Li, Qi Wu, Shengrong Sun

**Affiliations:** 1 Department of Breast and Thyroid Surgery, Renmin Hospital of Wuhan University, Wuhan, Hubei, P. R. China.; 2 Department of Oncology, Shanghai Artemed Hospital, Shanghai, P. R. China.; 3 Department of Orthopedics, Affiliated Provincial Hospital of Anhui Medical University, Hefei, Anhui, P. R. China.; 4 Tongji University Cancer Center, Shanghai Tenth People's Hospital, School of Medicine, Tongji University, Shanghai, P. R. China.

**Keywords:** microbiota, immunotherapy, fecal microbiota transplantation, antibiotic

## Abstract

The role of the microbiome in immunotherapy has recently garnered substantial attention, with molecular studies and clinical trials providing emerging evidence on the pivotal influence of the microbiota in enhancing therapeutic outcomes via immune response modulation. However, the impact of microbial communities can considerably vary across individuals and different immunotherapeutic approaches, posing prominent challenges in harnessing their potential. In this comprehensive review, we outline the current research applications in tumor immunotherapy and delve into the possible mechanisms through which immune function is influenced by microbial communities in various body sites, encompassing those in the gut, extraintestinal barrier, and intratumoral environment. Furthermore, we discuss the effects of diverse microbiome-based strategies, including probiotics, prebiotics, fecal microbiota transplantation, and the targeted modulation of specific microbial taxa, and antibiotic treatments on cancer immunotherapy. All these strategies potentially have a profound impact on immunotherapy and pave the way for personalized therapeutic approaches and predictive biomarkers.

## Introduction

Immunotherapy has ushered in a transformative era in oncological interventions, achieving significant advancements in this discipline 1. Approaches such as anti-programmed cell death-1 (PD-1) antibodies 2 and chimeric antigen receptor (CAR)-T cell therapy^3^ can rejuvenate the immune milieu and hinder the proliferation and dissemination of malignant cells. These therapies have been found to greatly enhance the survival rate and overall well-being of a substantial cohort of patients with cancer. However, patients undergoing immunotherapy often exhibit variable responses to such therapies, which can be partly attributed to the intricate interactions within the tumor immune microenvironment 1-3.

Researchers are increasingly acknowledging the pivotal role of the microbiota in regulating the immune system 4. The human microbiota is a diverse ecosystem comprising varied microorganisms, including bacteria, fungi, and viruses, which predominantly inhabit body sites such as the gastrointestinal tract, skin, and oral cavity. These microbiota engage in complex and nuanced interactions with the immune system to potentially influence the nature and magnitude of immune responses, thereby leading to a profound impact on the effectiveness of tumor immunotherapy 4.

Moreover, this relationship between the microbiota and the immune system has prompted comprehensive exploration into microbiome-based strategies for optimizing the efficacy of tumor immunotherapy 5. Various interventions, such as probiotics, prebiotics, fecal microbiota transplantation (FMT), and antibiotics, have been identified as potential tools for directly modulating the microbiome and its influence on immune responses 6-9. Therefore, altering human microbiota composition via these interventions is a promising approach for regulating the complicated interactions within the tumor immune microenvironment and improving immunotherapy effectiveness.

This comprehensive review aims to elaborate on the numerous immunotherapy applications in oncology and explore the complex interplay between microbiota and tumor immunotherapy. Moreover, we attempt to examine the influence of microbiota on the immune system and discuss the potential associations of microbiome-based therapies and antibiotics with tumor immunotherapy. Lastly, based on our extensive investigations into these aspects, we hope to provide further insights to facilitate the development of personalized strategies for tumor immunotherapy, ultimately enhancing treatment outcomes for patients with cancer.

## 1. Tumor immunotherapy development and historical findings on the impact of bacteria on immunotherapy

**Figure 1:**
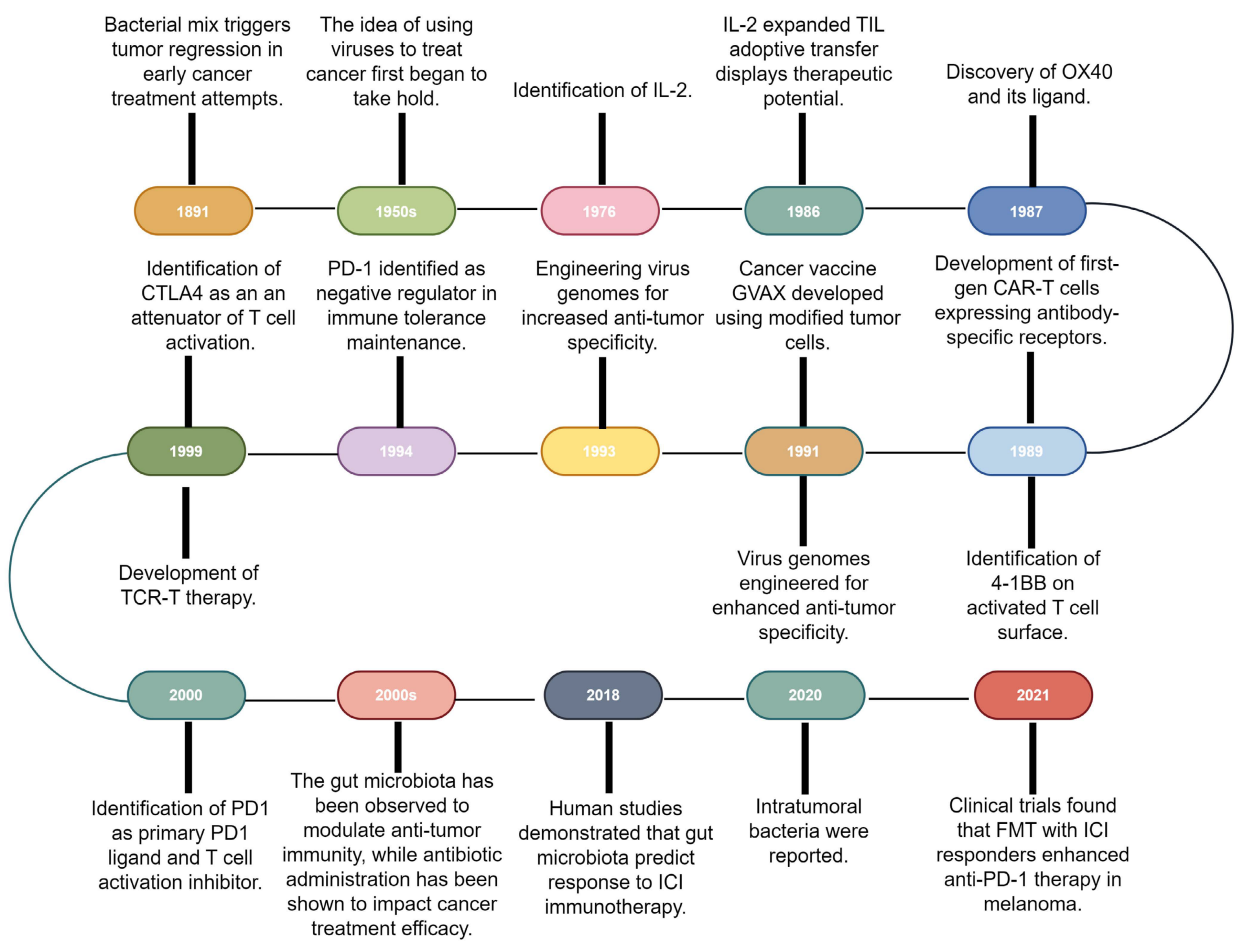
** Research history of immunotherapy and the role of microbiota in immunotherapy.** The timeline of immunotherapy advancements spans over a century, starting with William Coley's work in 1891. Milestones include IL-2 identification in 1976, OX40 receptor characterization in 1987, and first-gen CAR-T cells in the late 1980s. Other breakthroughs include virus genome engineering, MAGE-encoded antigen identification, CTLA4 discovery in the late 1990s, and PD-1/PD-L1 recognition as immune regulators. Gut microbiota's impact on anti-tumor immunity and antibiotic influence emerged, with 2018 studies linking microbiota diversity to ICI therapy response. In 2020, intratumoral bacteria presence was discovered, deepening knowledge of microbiota's role in cancer immunotherapy. 2021 trials showcased FMT's potential with anti-PD-1 therapy in melanoma patients. IL-2: Interleukin-2; CAR-T: Chimeric antigen receptor T-cell; CTLA4: Cytotoxic T-lymphocyte-associated antigen 4; FMT: Fecal microbiota transplantation; GVAX: GM-CSF gene transduced autologous tumor vaccine; ICI: Immune checkpoint inhibitor; PD-1: Programmed cell death-1; TCR-T: T cell receptor-engineered T cell; TIL: Tumor-infiltrating lymphocyte.

Immunotherapy, which primarily aims to enhance the innate immune mechanisms against malignant cells, is a groundbreaking advancement in cancer therapeutics and catalyzes a paradigm shift in oncology. Although the concept of leveraging the host immune system for eradicating cancer has existed for a century 10, remarkable progress and transformative developments in fundamental scientific investigations and clinical research endeavors have only been recently achieved 1. This field has genuinely succeeded in only recent years via significant advancements across a spectrum of therapeutic techniques, including oncolytic viruses, cancer vaccines, cytokine therapy, adoptive cell therapy (ACT), immune checkpoint inhibitors (ICIs), and immune-agonists 11-15. One intriguing aspect contributing to the progress in tumor immunotherapy is the historical findings related to the impact of bacteria on this therapeutic approach 16. Recent research has shed light on the intricate interplay between bacteria and the immune system, highlighting their potential roles in modulating immunotherapy response 17,18. These observations have added a new dimension to our understanding of immunotherapy, resulting in the influence of bacteria becoming increasingly recognized and integrated into the broader landscape of cancer treatment strategies (Fig. 1).

### 1.1 Oncolytic virus therapy

A cornerstone of the historical development of immunotherapy can be traced back to the pioneering observations of Virchow in 1863, who identified white blood cells of the immune system in tumor tissues, hinting at the intricate connection between inflammation and tumorigenesis 10. Subsequently, the visionary William Coley initiated a remarkable research direction in 1891 by experimenting with a mixture of live and inactivated cultures of pyogenic streptococci and *Bacillus* 19. This groundbreaking approach suggested the potential cancer-combating role of the immune system; however, the unknown mechanisms and inherent infection risks hampered the progress of this research perspective.

In the ensuing decades, oncolytic virus therapy has emerged as a beacon of hope. In this radical endeavor, a novel strategy harnessing the latent capabilities of meticulously designed genetically modified viruses that specifically targeted neoplastic cells was employed. This precise intervention initiates a pro-inflammatory microenvironment, which amplifies the systemic anti-tumor immune responses 20. Recent advancements in genetic engineering and viral manipulation methodologies have further thrust oncolytic virus therapy into the forefront of cancer treatment research. One such example of this advancement is the application of talimogene laherparepvec, commonly referred to as T-VEC or Imlygic, an innovatively engineered herpes simplex virus 21. T-VEC has demonstrated remarkable clinical efficacy, particularly in patients with advanced melanoma 21,22. In 2015, the United States Food and Drug Administration (FDA) approved the use of T-VEC, representing a significant milestone in oncolytic virotherapy. This approval, which was provided to T-VEC for managing the challenging clinical condition of metastatic melanoma, was a pivotal moment. Moreover, T-VEC is currently the only oncolytic virus immunotherapy sanctioned by the FDA. This revolutionary therapy is a second-generation approach that utilizes the herpes simplex virus type 1 (HSV-1) for treating metastatic melanoma. T-VEC strategically includes granulocyte-macrophage colony-stimulating factor (GM-CSF), a cytokine that is critical in immune system modulation.

The other oncolytic virus candidates in clinical trials cover a broad spectrum of solid tumors 23,24. Among them, coxsackievirus A21 is a naturally occurring virus that targets intercellular adhesion molecule-1. It exhibited well-tolerated characteristics when administered in conjunction with pembrolizumab in a phase Ib clinical trial. Furthermore, this combination treatment has been found to partially upregulate the expression of programmed cell death ligand-1 (PD-L1) on tumor cells 24. Another virus candidate, DNX-2401, also known as Delta-24-RGD, is an engineered adenovirus tailored for preferential replication within cells exhibiting Rb defects. A phase I trial showed promising clinical responses in 20% of patients with recurrent malignant glioma following the intratumoral injection of DNX-2401^25^. The viruses predominantly utilized in current clinical trials for oncolytic virus-mediated tumor therapy encompass adenoviruses, HSV-1, and poxviruses, reflecting the growing knowledge of deoxyribonucleic acid (DNA)-based viral agents 26. Additionally, a type 3 deattenuated strain of the orthoreovirus, which is from the family of non-enveloped double-stranded ribonucleic acid (RNA) viruses known as reoviruses, has been extensively investigated as an oncolytic agent 27.

Previous studies have also demonstrated that combining oncolytic viruses with conventional clinical anti-cancer therapies can enhance therapeutic efficacy. For example, integrating the measles virus with conventional chemotherapeutic agents, such as gemcitabine, has been reported to facilitate the lysis of senescent cancer cells across diverse tumor types 28,29. A phase III clinical trial involving the synergistic administration of PD-1/PD-L1 blockade and TG4010 (an engineered vaccinia virus Ankara) revealed markedly enhanced treatment efficacy among patients with advanced cancer 30,31. A study by Nishio *et al.* demonstrated that CAR-T cells armed with adenovirus expressing chemokine (C-C motif) ligand 5 (CCL5, better known as RANTES) and cytokine interleukin (IL)-15, specifically augmented CAR-T cell migration and proliferation in a xenotransplant human neuroblastoma murine model, ultimately leading to enhanced tumor-bearing mice survival 32.

### 1.2 Tumor vaccines

The elucidation of the immune system evasion mechanisms of cancer cells has resulted in the multifaceted advancement of cancer immunotherapy. This progress entails a broad spectrum of resources, including antibodies, peptides, proteins, nucleic acids, and immunocompetent cells, collectively contributing to the ongoing development of cancer immunotherapy 33. In the case of cancer vaccines, vaccination strategies can be grouped into three major categories according to their format and composition, i.e., protein-based vaccines, cell-based vaccines (encompassing immune or tumor cells), and nucleic acid-based vaccines.

The domain of cancer vaccines has heralded a paradigm change by utilizing tumor-specific antigens to provoke T cell-mediated immune responses that target tumors. One such pioneering research has identified the potential application of MZ2-E and MZ2-D, derivatives of melanoma-associated antigen gene family-encoded antigens with specific relevance to melanoma. These antigens have been shown to trigger cytotoxic T cells, inciting robust anti-tumor immune responses 11,12. Furthermore, the melanoma antigen gp100 has emerged as a pivotal factor in directing *in vivo* tumor rejection responses via immune reactions facilitated by tumor-infiltrating lymphocytes (TILs) in individuals with melanoma 34. All these findings have laid the groundwork for establishing tumor antigens as the cornerstone of cancer immunotherapy.

Autologous tumor cell vaccines using cells are one of the vaccine strategies currently under evaluation. Of these approaches, dendritic cell (DC)-based vaccination has emerged as a potent and promising strategy. DCs are often considered the quintessential antigen-presenting cells (APCs) with a central role in the anti-tumor immune responses. After activation by tumor antigens, DCs swiftly engulf, process, and present the resulting epitopes to T cells, eliciting cytotoxic T lymphocyte (CTL) responses. The development of DC-based vaccines involves the isolation of DCs, pulsing them with tumor antigens or tumor cell lysates, and ex vivo stimulation using precisely defined maturation cocktails 35. A prominent example of this approach is sipuleucel-T, a DC-based immunotherapy that has received approval for the treatment of advanced prostate cancer 36. Furthermore, using whole tumor cells as a therapeutic modality has been gaining momentum. The introduction of GM-CSF gene transduced autologous tumor vaccine (GVAX), an immunotherapeutic vaccine designed from autologous tumor cells that have been genetically modified to produce GM-CSF, has demonstrated significant potential in enhancing tumor-specific immune responses in a diverse array of cancer malignancies 37-39. All these advancements underscore the profound impact of tumor vaccines on the clinical landscape of cancer therapy.

DNA vaccines have also displayed promise in several initial studies 40,41. Among them, VGX3100, a DNA vaccine for cervical cancer, is currently undergoing phase 3 clinical trials 42. A total of 53 (49.5%) of 107 patients administered VGX-3100 exhibited histopathological regression. This outcome highlights the potential of VGX-3100 as a non-surgical therapeutic alternative for cervical intraepithelial neoplasia 2/3 (CIN2/3), potentially reshaping the treatment field for this prevalent ailment. In contrast to DNA vaccines, RNA vaccines do not integrate into the genome, mitigating concerns of carcinogenicity 43. Additionally, RNA vaccines function within the cytoplasm, thereby bypassing the need for nuclear entry 44. Consequently, the clearance of RNA vaccines is rapid, leading to minimal side effects. Although RNA is inherently less stable than DNA, various modifications, including formulations with liposomes or stabilizing adjuvants, can help enhance its stability 45-47. Other techniques have also been devised to fortify the RNA molecule, such as incorporating a 5′ cap structure, untranslated regions, and optimized codon usage within translated regions 48. Thus, continued advancement in nucleic acid delivery methods is a promising step toward the transformative development of the field of nucleic acid vaccines.

### 1.3 Cytokine therapy

Cytokines are versatile messengers within the complex immune network and are pivotal in modulating immune responses 49. Of these, IL-2 is a critical cytokine, originally regarded as a T-cell growth factor 50. IL-2 possesses a remarkable capacity for T-cell expansion *in vitro* and *in vivo*, manifesting potent immunostimulatory properties 51. Furthermore, the clinical administration of high IL-2 doses has demonstrated compelling evidence of cancer regression in patients with metastatic malignancies 52,53.

Another prominent therapeutic cytokine in cancer treatment is interferon-alpha (IFN-α) 54. This multifaceted type I IFN has a dual role in tumor control. The first role consists of the direct elimination of tumor cells via the induction of senescence and apoptosis, whereas the second one includes enhancing the effectiveness of anti-tumor immune responses by stimulating DC maturation and augmenting T-cell cytotoxicity 55. Clinical investigations have also underscored the therapeutic efficacy of high-dose IFN-α in conditions such as chronic myeloid leukemia and melanoma 56,57.

Moreover, chemokine networks are often dysregulated in cancer, with chemokines being significantly involved in the neovascularization processes. Malignant cells also regularly exploit the chemotactic activity of chemokines 58. Consequently, targeting specific chemokines or their tumor receptors has a solid preclinical rationale 49. C-X-C chemokine receptor type 4 (CXCR4), a chemokine receptor overexpressed in >75% of cancers, is crucial for tumor cell proliferation, dissemination, and angiogenesis 59. CXCR4 antagonists have demonstrated efficacy in restricting tumor growth in various experimental murine models. Plerixafor is one of the most common CXCR4 antagonists used in clinical applications. It has received approval for mobilizing hematopoietic stem cells, particularly in patients with non-Hodgkin lymphoma or multiple myeloma 60.

Although cytokine therapy has potential clinical value, its practical adoption as a standalone treatment has been impeded by challenges concerning its tolerability and severe toxicity 49. Nevertheless, cytokines are still helpful when employed in conjunction with other immunotherapeutic approaches, particularly with ACT, effectively alleviating these obstacles.

### 1.4 ACT

ACT is a groundbreaking strategy that utilizes autologous immune cells, primarily T cells, natural killer (NK) cells, and macrophages. In this therapy, such cells are isolated, genetically modified, expanded ex vivo, and reintroduced into the patients. The primary objective of ACT is eliminating cancer cells and accomplishing sustained clinical effectiveness 13. The employment of highly selective, tumor-reactive T cells has emerged as a transformative strategy, particularly in ACT for patients with metastatic melanoma presenting with the characteristic overexpression of endogenous differentiation antigens 61. This approach has yielded sustained clonal expansion of T cells in patients with cancer. Moreover, the innovative use of genetically engineered and custom-designed T cells targeting novel antigens has gained considerable traction. Subsequently, two distinct categories of transgenic T cells with substantial contributions to the treatment of malignant tumors have been developed, namely CAR-T cells and T-cell receptor (TCR)-engineered T cells 62.

CAR-T cell therapy, which utilizes antibody fragments to precisely target the surface antigens of cancer cells, has undergone considerable development. The transition from first-generation CAR-T cells containing immunoglobulin-TCR chimeric receptors 63 to second-generation CARs incorporating co-stimulatory molecules such as the cluster of differentiation (CD) 28 marks a pivotal milestone in this progress 64. This transformation has enabled modified T cells to exhibit prolonged *in vivo* persistence. Furthermore, researchers have explored the effectiveness of combining other molecules within CARs, demonstrating success in the gene modification of autologous T cells to express an anti-CD4 antibody connected to CD19-zeta and 19-3BB signaling domains. This combination strategy in CARs has effectively induced immune responses in patients with chronic lymphocytic leukemia 65,66. All these findings stress the anti-tumor potential of CAR-T therapy across a range of human cancers.

T cells interact with peptides derived by means of a cell-surface receptor, the T-cell receptor (TCR), which is a disulfide-bonded heterodimeric protein composed of α and β chains. This receptor complex is augmented in functionality by associations with the CD3ε, γ, δ, and ζ subunits 67. Upon encountering peptides that have been processed and displayed on major histocompatibility complex (MHC) molecules, TCRs initiate signaling events that lead to T-cell activation. In humans, these antigen-presenting MHC molecules are categorized into human leukocyte antigen (HLA) class I and HLA class II, with the former typically presenting peptides from cytoplasmic sources and the latter from extracellular compartments 68. The engagement of the TCR with MHC molecules is facilitated by coreceptors CD8 and CD4, respectively, which are integral for enhancing the sensitivity of TCR-mediated antigen recognition 69. Upon TCR binding to its cognate MHC, a cascade of intracellular events is triggered, including the phosphorylation of immunoreceptor tyrosine-based activation motifs (ITAMs) within the CD3 subunits. This phosphorylation is instrumental in activating the T cell, leading to a suite of effector responses, such as cell proliferation, cytokine production, and the execution of cytotoxic functions mediated by perforin and granzyme release 70. The development of TCR-engineered T cells, commonly known as TCR-T cell therapy, can be attributed to the groundbreaking work by Clay *et al.* 71. This pioneering approach involved the transfer of TCR genes into peripheral blood lymphocytes obtained from patients with melanoma, ultimately leading to the ex vivo generation of tumor-reactive effector T cells. Subsequently, clinical validation was quickly attained, wherein patients with metastatic melanoma experienced tumor regression following TCR-T cell therapy 72. Moreover, transgenic TCR-T cells targeting the cancer-testis antigen NY-ESO-1 demonstrated sustained antigen-specific anti-tumor effects 73, eventually resulting in tumor regression. Hence, CAR-T cell and TCR-T cell therapies have made pronounced strides in cancer treatment, offering encouraging clinical outcomes 74,75. CAR T-cell therapy requires the incorporation of autologous T cells to address concerns related to allogeneic activity and graft-versus-host disease. However, many patients exhibit diminished peripheral blood T-cell counts due to extended pre-CAR-T treatment regimens. This situation substantially delays therapy and can render CAR-T manufacturing impractical. Additionally, the CAR-T manufacturing process is protracted and intricate, leading to an increasing number of patients becoming ineligible or experiencing disease progression post-treatment. A prior study revealed that 22.5% (16/71) of patients failed to achieve the targeted CD3^+^ T-cell yield for CAR-T production via autologous lymphapheresis 76.

Considering that NK cells can activate autonomously (regardless of the MHC pathway) and exhibit diminished alloreactivity risks, the generation of CAR-NK cells does not require autologous NK cells. The available resources, such as NK92 cell lines, umbilical cord blood, and induced pluripotent stem cells (iPSCs), can be utilized for this purpose 77. To date, most CARs employed in CAR-NK cell research have applied CAR architectures identical to those employed in CAR-T cells. These CAR structures typically contain either the same CD3ζ intracellular domain observed in first-generation CAR-T cells 78 or the CD3ξ co-stimulatory domain used in second-generation CAR-T cells, such as 4-1BB^79^. The inclusion of the 4-1BB co-stimulatory domain has been found to significantly enhance the activation and cytotoxic potential of NK cells as well as the secretion of key cytokines, including IFN-γ and GM-CSF. Li *et al.* conducted a comprehensive comparative analysis between a CAR-T cell construct and various CAR-NK cell variants to further refine the design of CAR-NK cells. Their results showed that CAR-NK cells had unique transmembrane and intracellular signaling domains that effectively targeted a specific antigen. Furthermore, CAR-NK cells with the NKG4D transmembrane domain, 2B4 co-stimulatory domain, and CD3ζ signaling domain demonstrated remarkable cytotoxicity that was robust and antigen-specific. CAR-NK cells derived from iPSCs harboring this specific construct also exhibited a characteristic NK cell phenotype, showing substantial anti-tumor activity and prolonged *in vivo* persistence 80.

More recently, CAR-macrophages have emerged as a highly promising and innovative therapeutic approach for cancer treatment 81. Compared to CAR-T and CAR-NK cells, CAR-macrophages offer unique and distinct advantages for addressing two critical challenges of managing solid tumors. These problems pertain to the trafficking and infiltration of immune cells into the tumor microenvironment (TME) and the amelioration of the immunosuppressive conditions within the TME 82. According to their functional properties and major activation states, macrophages can be classified into the following two groups: pro-inflammatory M1 and anti-inflammatory M2 macrophages. Among these, tumor-associated macrophages, particularly those of the M2 subtype, are widely acknowledged as a pivotal group of immunosuppressive cells within the TME 83. Despite their immunosuppressive effects on other immune cells, M2 macrophages retain their phagocytic capabilities, exhibiting a higher phagocytic activity than M1 macrophages. Moreover, macrophages possess a significant degree of phenotypic plasticity, enabling them to adapt to environmental cues and modify their phenotypes 84. CAR constructs employed in CAR-engineered macrophages display structural components similar to those observed in CAR-T cells. These structures consist of an extracellular antigen-binding domain, hinge region, transmembrane domain, and intracellular domain. Nevertheless, one distinctive feature of CAR-engineered macrophages is the composition of their intracellular signaling domains. Similar to CAR-T cells, CAR-macrophages utilize the CD3ζ intracellular domain, which incorporates ITAMs derived from immune receptor tyrosine kinases of the Src family 85-87. In CAR-T cells, the ITAMs undergo phosphorylation after CAR engagement by Src family kinases and subsequently interact with the tSH70 domain in zeta-chain-associated protein kinase 2 (ZAP2), culminating in CAR-T cell activation for cytotoxic function. In contrast, macrophages lack ZAP2 expression. Thus, they express the kinase Syk, which houses a tSH3 domain that can bind to CD100ζ, ultimately mediating phagocytic signaling in the macrophages 88. Currently, only one phase I clinical trial of CAR-macrophages has been officially registered (ClinicalTrials.gov identifier: NCT04660929). This clinical trial involves the CAR-macrophages originally developed by Klichinsky *et al.* In that approach, the researchers modified CAR-macrophages by integrating a hybrid adenovirus vector Ad5f35 and a specific targeting mechanism utilizing single-chain variable fragments against human epidermal growth factor receptor 2. The adenoviral infection triggers macrophage polarization toward a pro-inflammatory M1-like phenotype. However, no outcomes or findings of this clinical trial have been released since its initiation in 2021. Simultaneously, a second study involving CAR macrophages has also been registered (ClinicalTrials.gov identifier: NCT05007379). This observational study is designed to evaluate the anti-tumor activity of CAR-macrophages derived from the organotypic cultures of patients.

### 1.5 ICIs

**Figure 2:**
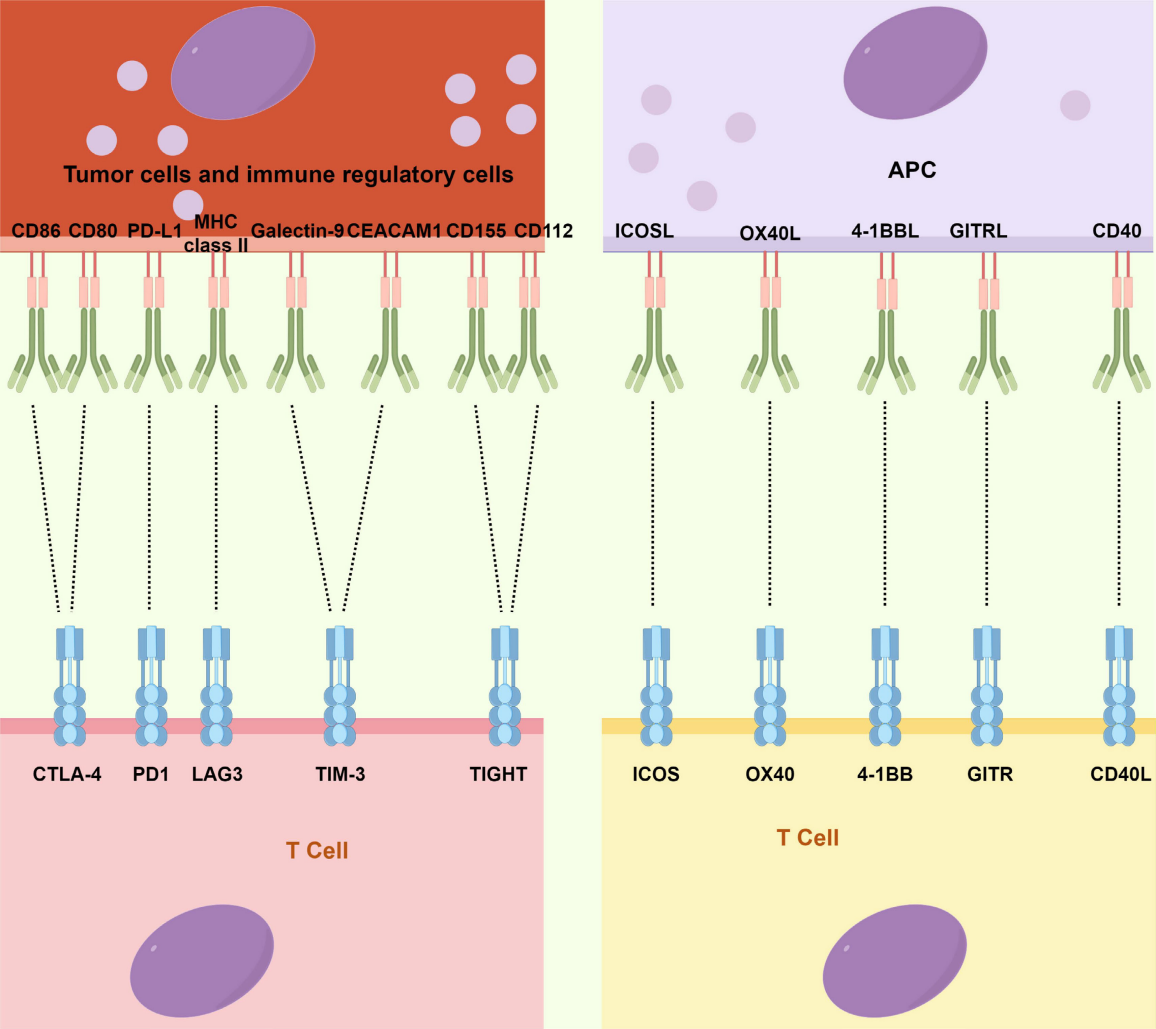
** The primary immune checkpoints and immune-agonists site on cancer cells and immune cells.** CEACAM1: Carcinoembryonic antigen cell adhesion molecule 1; CTLA4: Cytotoxic T-lymphocyte-associated antigen 4; GITR: Glucocorticoid-induced TNF receptor; ICOS: Inducible T-cell costimulator; LAG3: Lymphocyte-activation gene 3; MHC: Major histocompatibility complex; PD-1: Programmed cell death-1; PD-L1: Programmed cell death ligand-1; TIGHT: T-cell immunoglobulin and ITIM domain; TIM3: T-cell immunoglobulin and mucin domain-containing protein 3.

Although ACT has made significant advancements, the emergence of ICIs, a novel category of monoclonal antibodies, has taken center stage in the field of immunotherapy and has assumed a pivotal role in therapeutic interventions 14. Immune checkpoints, which constitute molecules participating in co-inhibitory signaling pathways, are critical in maintaining immune tolerance. However, these checkpoints are frequently co-opted by cancer cells to evade immune surveillance (Fig. 2). ICIs are specifically engineered to reinvigorate anti-tumor immune responses by disrupting these co-inhibitory signaling pathways, eventually facilitating the immune-mediated elimination of malignant cells 89,90. The primary targets of ICIs include cytotoxic T-lymphocyte-associated antigen 4 (CTLA4), PD-1, PD-L1, lymphocyte-activation gene 3 (LAG3), T-cell immunoglobulin and mucin domain-containing protein 3 (TIM3), and T-cell immunoglobulin and ITIM domain (TIGIT).

CTLA4 is an inhibitory molecule expressed on T cells and is crucial in modulating T-cell activation following the interaction of the MHC with the TCR 91,92. The primary function of CTLA4 is to preserve immune homeostasis by negatively regulating T-cell activation 93. This inhibitory activity of CTLA4 is attributed to its ability to outcompete the co-stimulatory receptor CD28 for binding to the B7 co-stimulatory ligands, CD80 and CD86, via its relatively higher binding affinity and avidity 94. Consequently, CTLA4 effectively dampens T-cell activation. Additionally, CTLA4 can reduce the surface expression of CD80 and CD86 on APCs via trans-endocytosis, thereby curtailing cytotoxic T-cell activation 95. The TME of various cancers has been shown to accumulate CD4^+^CD25^+^Foxp3^+^ regulatory T cells (Tregs), which also express elevated levels of CTLA4 96. The stabilization of CTLA4 expression by Foxp3, in turn, amplifies the attenuation of T-cell response in the context of cancer 97. Seminal research has further demonstrated that the antibody blocking of CTLA4 can evoke robust immune responses, leading to tumor regression 98. Subsequent clinical trials and efficacy assessments resulted in ipilimumab, a CTLA4-targeting monoclonal antibody, receiving approval as the first ICI for cancer treatment 99,100.

PD-1, originally linked to programmed cell death, is an immune checkpoint expressed on the T-cell surface 101. The mechanisms underlying PD-1 regulation remained unclear until the discovery of its ligand, PD-L1. This ligand is not only expressed in normal tissues but also exploited by tumor cells to evade immune surveillance 102. Moreover, the classical two-signal hypothesis has underscored the necessity of proper T-cell activation, which requires TCR and MHC engagement and ensuing co-stimulatory signals mediated by CD28 via the B7 ligands 103. Previous research has demonstrated that blocking the PD-1/PD-L1 pathway can rejuvenate the cytotoxic potential of T cells and elicit tumor regression, thus establishing PD-1 and PD-L1 as promising therapeutic targets 104,105. After the groundbreaking approval of pembrolizumab for advanced melanoma treatment in 2014, the clinical utility of PD-1/PD-L1 inhibitors has expanded to various cancer types, including head and neck squamous cell carcinoma 106-108, non-small cell lung cancer (NSCLC) 109, and renal cell carcinoma (RCC) 110. The widespread use of ICIs has also uncovered a range of independent immune-related adverse events. Some patients experience severe immune-related side effects, leading to complications such as pneumonia, myocarditis, or hepatitis 111,112. Additionally, a substantial proportion of patients exhibit primary resistance that arises from diverse mechanisms, including the inherent resistance of tumor cells to T-cell engagement, limited tumor immunogenicity, impaired CD8^+^ T-cell migration to the tumor site, and immune inhibitory factors, such as myeloid-derived suppressor cells (MDSCs) and Tregs, within the TME 2,111.

The LAG3 gene is expressed by T and NK cells after the ligation of MHC class II molecules 113. Although the precise mechanism remains elusive, LAG3 is suggested to be pivotal in negatively regulating T-cell function, thus protecting against tissue damage and autoimmunity. LAG3 is often co-expressed and upregulated on TILs along with PD-1, contributing to immune exhaustion and facilitating tumor growth 114. Consequently, LAG3 blockade was found to enhance anti-tumor immunity and complement other immunotherapeutic approaches via a distinct mechanism involving the inhibition of cell cycle progression 115. Although the concurrent administration of anti-LAG3 and anti-PD-1 therapies are considered synergistic, the effectiveness of combining anti-LAG3 therapy with other ICIs is still uncertain 116. Further, the clinical benefits of such combination therapies are potentially accompanied by an increased occurrence of autoimmune toxicities 117.

TIM3 is a crucial direct negative regulator of T cells and is also present in NK cells and macrophages 118. This receptor influences the immunosuppressive milieu indirectly, chiefly by promoting MDSC expansion 119. The heightened TIM3 levels are closely correlated with T-cell dysfunction and exhaustion, emphasizing its pivotal role in various malignancies. In particular, the presence of TIM3-expressing T cells in NSCLC and follicular lymphoma is closely associated with disease severity and an unfavorable prognosis 120. Conversely, diminished TIM3 levels have been linked to autoimmune conditions, such as diabetes and multiple sclerosis 119. The intervention of TIM3 blockade via monoclonal antibodies may enhance T-cell proliferation and cytokine production, thereby indicating its potential anti-tumor efficacy and simultaneous risk of exacerbating autoimmune diseases. Therefore, the application of these antibodies is concerning because certain acute infections, including *Listeria* infections, may be aggravated by TIM3-mediated CD8^+^ T-cell enhancement 121. Additionally, several ligands, such as galectin-9, phosphatidylserine, and carcinoembryonic antigen cell adhesion molecule 1, have been reported to modulate the TIM3 pathway 120. These ligands are vital for carcinogenesis, tumor survival, and the progression of various malignancies, including melanoma, gastrointestinal cancer, and lung cancer 122-124. In contrast to other immune inhibitory pathways that interfere with cellular function, TIM3 mainly modulates cell apoptosis. This feature may explain its augmented effects when combined with other ICIs 118. However, the optimal molecule that can be combined with TIM3 is yet to be identified.

TIGIT is a CD28 family-like receptor expressed on NK and T cells. This receptor directly suppresses immune responses and indirectly regulates the release of immunoregulatory cytokines, including IL-10. TIGIT also diminishes IFN-γ and IL-17 production and impedes DC maturation 125. The two agonists of TIGIT, CD155 (also known as poliovirus receptor [PVR]) and CD112 (alternatively termed as PVRL2 or nectin-2), are widely expressed by immune, non-immune, and tumor cells, including melanoma cells 126. TILs often co-express high levels of TIGIT as well as PD-1, TIM3, and LAG3, indicating a dysfunctional phenotype 127. Preliminary studies have shown that simultaneously blocking TIGIT and either PD-1 or TIM3 promotes immune cell proliferation, cytokine release, and degranulation and reverses T-cell exhaustion, which in turn results in tumor rejection and protective memory responses 120,128. Moreover, TIGIT expression is more prominent in the TME than in peripheral cells, thereby allowing more precise and less toxic therapy. Additionally, TIGIT mainly alters cytokine production and CD8 T-cell function, suggesting its complementary role with other ICIs 129.

### 1.6 Immune agonists

Checkpoint inhibitors have led to notable success in cancer treatment. However, over 80% of patients do not achieve a favorable response after ICI treatment or develop resistance over time. Consequently, research efforts have noticeably shifted toward enhancing anti-tumor activity by manipulating signaling pathways with agonistic antibodies targeting specific molecules to augment anti-tumor T-cell responses 15. The comprehensive activation of T cells requires a trio of signals, including TCR signaling, co-stimulatory signaling, and cytokine support 130. TCR signaling primarily revolves around the recognition of neoantigens, which exhibit distinct expression patterns exclusive to tumor cells. These neoantigens originate from genetic mutations within tumor cells, resulting in peptide epitopes distinct from those originating from the standard human genome 131. The neoantigen peptide-MHCs are then conspicuously displayed on the surfaces of tumor cells and APCs, providing a strong target for the TCRs on antigen-specific T cells 132. Furthermore, interventions focused on regulating TCR signaling, such as the pioneering approach of CAR-T therapy, have already proven their worth in clinical applications 133.

Various co-stimulatory pathways are involved in T-cell activation 134. One key co-stimulatory cascade that significantly contributes to T-cell activation and cytokine release is the CD80/CD86-CD28 signaling pathway 135. Conversely, T-cell suppression occurs due to the relatively higher binding affinity of CTLA4 for CD80/CD86 136. Another inducible co-stimulatory receptor expressed on activated T cells is the inducible T-cell co-stimulator (ICOS), which interacts with the ICOS ligand 137. Apart from these co-stimulatory receptors, receptors such as OX40, 4-1BB, glucocorticoid-induced tumor necrosis factor (TNF) receptor (GITR), and other TNF superfamily members can synergize with TCR signaling, ultimately enhancing T-cell responses and survival. Additionally, alternative mechanisms for boosting the anti-tumor responses of T cells involve the co-stimulatory receptors, such as CD40, on APCs 138.

ICOS, a member of the CD28 superfamily, undergoes rapid induction following T-cell activation to deliver secondary co-stimulatory signals 139,140. Moreover, its ligand, known as the ICOS ligand, is predominantly expressed on B cells, macrophages, and DCs. The ICOS ligand-ICOS signaling pathway moderately facilitates T-cell proliferation, augments the production of cytokines (including IL-4, IL-5, IL-10, IFN-γ, and TNF-α), initiates Foxp3 transcription, and represses Tregs 141. Although the humanized ICOS agonist JTX2011 has delivered encouraging results in murine cancer models, it has shown limited efficacy when administered as monotherapy in clinical trials 142,143. Nevertheless, its use in conjunction with other checkpoint inhibitors has yielded slightly more promising results.

OX40 (also known as CD134) is a member of the TNFRSF4 family and is predominantly expressed on activated CD4/CD8^+^ T cells and Foxp3CD4 Tregs, especially within the intratumoral Treg population. Moreover, OX40 expression is transient, peaking at 24-48 h post-activation and persisting for 3-4 days 144. The ligand of OX40, CD252 (also designated as OX40 ligand [OX40L]), is found on activated APCs, including DCs, B cells, and macrophages 144,145. The OX40-OX40L signaling pathway enhances effector T-cell expansion and survival, eventually promoting memory T cells and inhibiting Tregs 146. Prior clinical trials have demonstrated that combining OX40 agonists with PD-1 blockade improves treatment outcomes in murine models; however, the results in patients were less favorable 147.

The co-stimulatory receptor 4-1BB is expressed on CD4^+^ and CD8^+^ T cells in murine models, whereas its expression in humans is predominantly in activated CD8^+^ T cells, NK cells, NKT cells, Tregs, DCs, and various myeloid cells. The cognate ligand, 4-1BBL, shows inducible expression patterns on activated APCs, myeloid progenitors, and hematopoietic stem cells. After its activation, 4-1BB engages with CD137L to initiate the activation of multiple signaling pathways, including nuclear factor κappa-B (NF-κB), extracellular signal-regulated protein kinases, c-Jun N-terminal kinase, and mitogen-activated protein kinase pathways, culminating in increased cytotoxic T-cell activity 148. Nevertheless, the role of 4-1BB in the regulation of Tregs is complex and context-dependent. Multiple 4-1BB-targeting agonists have been developed, which have the potential to enhance T-cell persistence, functionality, and expansion 149. Clinical trials have also explored the combination of 4-1BB agonists with immunotherapy or chemotherapy and yielded diverse outcomes, encompassing heightened anti-tumor efficacy and improved survival rates in select cases 150-152.

GITR (also termed CD357) is a member of the TNF receptor superfamily 18 and exhibits robust expression on Tregs, thus wielding a significant influence on their expansion and differentiation 153. GITR is expressed at lower levels on naive and memory T cells 154, while its ligand, the GITR ligand, has limited expression in APCs, including DCs, macrophages, and B cells. GITR is also pivotal in enhancing T-lymphocyte activity via the upregulation of IL-2 and IFN-γ and the concurrent inhibition of TCR activation-induced apoptosis, thereby aiding T-cell survival 155-157. Preclinical investigations involving GITR agonists in murine models have showcased their effectiveness in promoting the activation of CD4^+^ and CD8^+^ T cells and the suppression of intratumoral Tregs 158. Early-phase clinical trials centered on GITR agonists have also reported favorable safety profiles, with certain patients displaying sustained disease stability 159.

CD40, a constituent of the TNFR superfamily 5, is expressed on various immune cell types, including DCs, B cells, monocytes, and vascular/epithelial cells. Additionally, its ligand, CD40L (also known as CD154), is transitorily expressed on activated T cells 160. CD40 signaling in DCs is crucial in promoting the release of cytokines, upregulation of co-stimulatory molecules, cross-presentation of antigens, and modulation of pro- and anti-apoptotic gene expression 161. CD40 ligation can also induce apoptosis in some malignancies 162, while CD40-activated macrophages have been shown to exhibit tumoricidal activity, deplete tumor stroma, and induce tumor regression, all independently of T cells 163. Clinical trials investigating CD40 agonists have demonstrated good safety and objective responses in some patients, with combination therapies incorporating CD40 agonists also showing promise in overcoming immune refractoriness 164-166.

### 1.7 Bacterial insights in immunotherapy history

In the first decade of the 21st century, a notable advancement was achieved in understanding the modulation of CD8 T-cell activity by the gut microbiota and the consequential stimulation of anti-tumor immune responses 167. Furthermore, the efficacy of cancer treatments was reported to be compromised in the presence of antibiotics or germ-free mouse models, with these observations being contingent upon the specific species within the gut microbial community. In a seminal investigation in 2015, a groundbreaking correlation was established between the gut microbiota and ICI responses in murine models 168,169. The research underscored the impact of gut microbiota composition on anti-PD-L1 therapy response and showed that FMT could mitigate the response discrepancies. Moreover, the oral administration of *Bifidobacterium* was revealed to facilitate DC maturation, thereby enhancing the initiation and accumulation of CD8 T cells in the TME. Conversely, this restoration of anti-tumor efficacy was impaired by PD-L1 blockade 168.

Parallel studies of anti-CTLA4 therapy have indicated that antibiotics suppress the anti-tumor effects produced by ICIs. In contrast, supplementation with susceptible *Bacteroides fragilis* in germ-free or antibiotic-treated melanoma mice accentuated the therapeutic effects of anti-CTLA4. Furthermore, the microbiota-dependent anti-tumor effects were found to be intricately linked to the induction of Th1 cell activation in tumor-draining lymph nodes (LNs) and the maturation of intratumoral DCs 169. A series of human studies published in 2018 collectively proposed that the composition and diversity of the gut microbiota might serve as predictive indicators of ICI immunotherapy responses 170,171.

In line with this notion, FMT from ICI-responsive patients to germ-free or antibiotic-treated mice ameliorated tumor control and heightened ICI responses, whereas FMT from non-responders failed to yield these beneficial effects 163,164. Patients with NSCLC or RCC who exhibited elevated gut bacterial diversity also demonstrated heightened sensitivity to anti-PD-1 therapy 170. Similarly, an investigation involving patients with metastatic melanoma detected elevated baseline levels of *Bifidobacterium longum*, *Collinsella aerofaciens*, and *Faecalibacterium prausnitzii* in the feces of responder patients 164. Prospective studies conducted between 2019 and 2020 have further confirmed the noteworthy correlation between the gut microbiota of patients with NSCLC, hepatocellular carcinoma, or RCC and ICI outcomes 172-175. In 2020, two extensive studies examined the diverse intratumoral microbiota across more than 30 cancer types. Poore *et al.* researched the varied microbial communities within tumors and proposed a novel diagnostic tool based on the cancer-associated microbiota 176. Subsequently, Ravid *et al.* comprehensively analyzed seven tumor microbiome profiles and presented imaging evidence of the spatial distribution and intracellular localization of these microbial communities within tumors 177. A surprising finding from a 2021 clinical trial indicated that FMT from ICI responders coupled with anti-PD-1 therapy enabled patients with melanoma to overcome their resistance to PD-1 blockade therapy 8. Therefore, a growing body of evidence in the current landscape of tumor immunotherapy research underscores the significance of the complex and diverse gut microbiota in modulating immune-based treatment outcomes.

## 2. Microbiota impact on cancer immunity

The human microbial community primarily resides within the gastrointestinal tract, followed by other physiological interfaces between the human body and the external environment. These microbial communities predominantly orchestrate diverse regulatory effects on various host functions via immunomodulation 178. Additionally, recent research findings underline the substantial contribution of intratumoral microbiota to cancer immunodynamics 179. Microbes residing at distinct anatomical locations can also have varying impacts on systemic immunity and the immune milieu within tumors. Thus, a comprehensive exploration of this complex interplay between the microbial community and immune responses continues to be a focal point of contemporary research.

### 2.1 The intricate relationship between gut microbiota and tumors

#### 2.1.1 Influence of gut microbiota on adaptive immune responses

**Figure 3:**
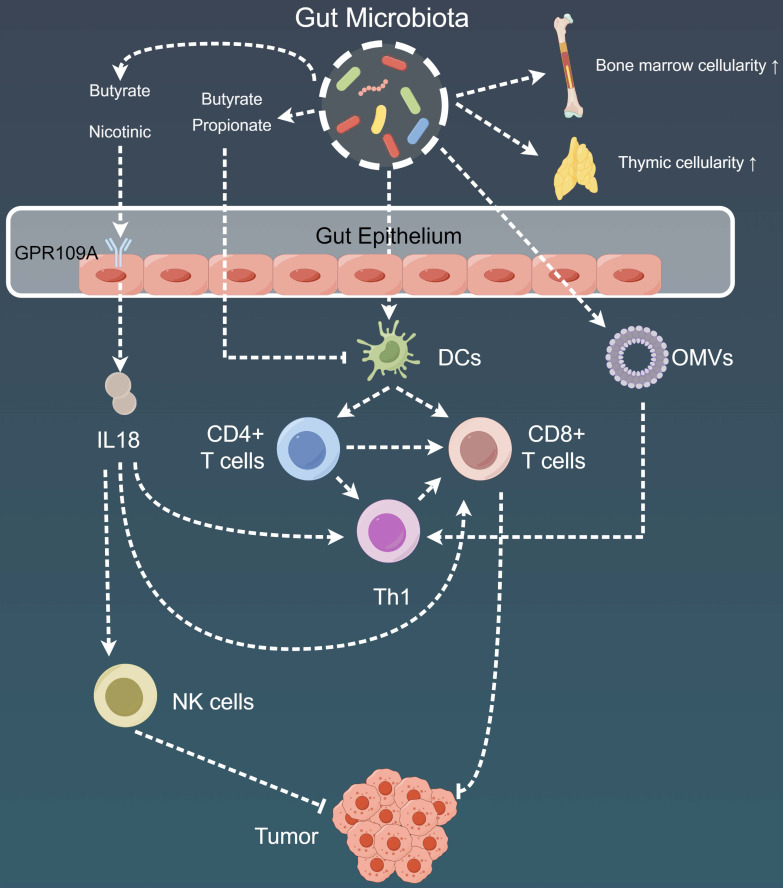
** Mechanistic insights into the impact of gut microbiota on tumor immunity.** Gut microbiota play a pivotal role in modulating adaptive immune responses through DCs present in the gut-associated lymphoid tissues, spleen, or tumor-draining lymph nodes. Additionally, metabolites produced by gut microbiota, such as butyrate and propionate, exert effects on regulating immune cell activation and resistance to certain immunotherapies. Furthermore, the gut microbial community impacts the systemic immune response by influencing bone marrow cellularity and thymic cellularity. Moreover, the intricate effects of the gut microbiome extend to its influence on the local and distant tumor microenvironment, orchestrating a complex interplay. DC: Dendritic cell; GPR109A: G-coupled receptor-109a; NK: Natural killer; OMV: Outer membrane vesicles.

The emerging body of evidence has emphasized the complicated connections between anti-cancer therapies and specific commensal-driven immune responses, thereby contributing to a deeper understanding of their mechanistic foundations (Fig. 3, Table 1) 180,181. Some notable associations include the promotion of *Enterococcus gallinarum* translocation following cyclophosphamide treatment, which results in pathogenic T helper cell (Th)17 responses and the production of IFN-producing CD8 T effector cells. This coordinated immune response effectively restrains tumor growth in sarcoma and lung adenocarcinoma models 182,183. Another important connection is the increased presence of *B. fragilis* and *Bifidobacterium* species in the fecal samples of patients with melanoma who received CTLA4 blockade. Subsequently, this enrichment activates toll-like receptor 4 (TLR4) and IL-12-dependent Th1 responses, contributing to therapeutic effectiveness 169. Studies have also detected a prominent relationship, wherein the effectiveness of PD-L1 inhibition in eliciting T-cell responses against melanoma is significantly enhanced in hosts harboring *Bifidobacterium* species within their microbiota 168,171. Lastly, oxaliplatin-induced ileal enterocyte death has been demonstrated to be a pivotal factor regulating the balance between anti-tumor follicular T helper (TFH) cells and potentially deleterious Th17 cells in colon cancer. This balance is finely modulated by the ratio of immunogenic *Enterococcaceae* to tolerogenic *Lactobacillaceae* 184.

Moreover, in most of these models, DCs derived from gut-associated lymphoid tissues (GALTs), spleen, or tumor-draining LNs played a critical role as intermediaries, with the DCs actively sensing various commensal microorganisms, including *Bacteroides*, *B. fragilis*, Mu*cispirillum*, *Ruminococcaceae*, and *Rodentibacter*. These interactions further triggered immune responses via the IFN-I and IL-12-mediated pathways 168,169,185-187. In addition to their role as DC adjuvants, the gut microbes are a rich source of antigens capable of eliciting commensal-specific T-cell responses on a systemic level. These responses can either exert detrimental or protective effects on the host, depending on the specific peptides involved.

A study by Gil-Cruz *et al.* elucidated how shared structural features between a β-galactosidase enzyme derived from* Bacteroides thetaiotaomicron* and host cardiac myosin heavy chain-6 could instigate a devastating autoimmune myocarditis condition 188. In contrast, Nanjundappa *et al.* indicated that cross-reactivity between an integrase enzyme obtained from *Prevotella* and the host islet-specific glucose-6-phosphatase catalytic subunit-related protein could redirect autoreactive CD8 T cells, thus ameliorating colitis 189.

Recent investigations have improved our understanding of cross-reactive homologs and enabled the inclusion of exogenous dietary antigens, particularly in the case of human leukocyte antigen-DQ2.5-mediated celiac disease 190. Molecular simulation studies have offered insights into the interplay between cancer and microbial antigens 191,192, wherein the concept of molecular mimicry between cancer and microbial antigens has been explored in-depth. Specifically, immune responses mediated by H-2Kb-restricted T cells against a spectrum of enterococcal bacteriophages exhibited cross-reactivity with an oncogenic driver factor, the proteasome 20S subunit beta 4 (PSMB4). Oral administration of bacteria carrying these bacteriophages led to enhanced bacteriophage-specific T-cell responses during cyclophosphamide or anti-PD4 antibody treatment, effectively countering extraintestinal tumors overexpressing PSMB4^192^. Correspondingly, T cells targeting a specific peptide expressed in the commensal bacterium *Bifidobacterium* showed a cross-reaction with a model neoantigen expressed in mouse melanoma B16-SIY 193. Furthermore, some human T cells with specificity for naturally processed melanoma epitopes showed recognition of microbial peptides, implying its clinical relevance 192.

Nevertheless, mechanisms other than molecular mimicry may also be involved in enhancing anti-tumor immunity. For instance, Tanoue *et al.* revealed an interesting consortium of 11 bacteria that could promote tumor antigen-specific CD8 IFN-γ T-cell responses during ICI. Moreover, these responses demonstrated no cross-reactivity with microbial antigens and did not originate from the colon. All these findings collectively highlight the intricate interplay between the gut microbiota and host immune responses in the context of cancer, thereby providing novel perspectives on potential avenues for therapeutic intervention 194.

Tumor irradiation has been proven to be significantly boosted in the presence of vancomycin, which eradicates the immunosuppressive metabolites, especially butyrate and propionate, originating from *Clostridia* 185,195. This elimination process is suggested to function via the elevated presentation of DC antigen and the concurrent priming of CD8 T cells. Conversely, metabolites derived from the gut microbiota, such as propionate, and those associated with the tryptophan pathway (+1H-indole-3-aldehyde and quinolinic acid), have been shown to confer long-term protection against radiation *in vivo* 196.

Moreover, increased butyrate and propionate concentrations have been linked to CTLA4 blockade resistance in murine models and patients with melanoma. This resistance is accompanied by an increased proportion of Tregs, diminished activation of DCs and effector T cells, and weakened responses to IL-2^197^. These metabolites have also been found to correlate with extended progression-free survival (PFS) after anti-PD-1 therapy 198.

Prebiotic inulin-type fructans have also been found to act as potent modulators of the gut microbiota, promoting the *in vitro* growth of *Bacteroides* species. This modulation mechanism exerts a restraining effect on the growth kinetics of the gut microbiota, effectively curbing T cell-dependent invasive melanoma 199. The action of prebiotic inulin occurs via distinct mechanisms, including facilitating the dominance of *Bacteroides* species in the gut microbiota, improving CTL function within the spleen, and successfully combating melanoma resistance to mitogen-activated extracellular signal-regulated kinase inhibitors 199. This sophisticated interplay between the gut microbiota, immunosuppressive metabolites, and therapeutic interventions underlines the intricate landscape of tumor immune responses and opens up promising avenues for therapeutic exploration.

#### 2.1.2 Significance of gut microbiota in systemic immunity

The establishment of a resilient immune system is of paramount significance in the context of allogeneic hematopoietic stem cell transplantation (HSCT), exhibiting a critical role in relapse management and the reduction of transplant-associated mortality in patients 200,201. Recent extensive clinical trials conducted across various centers and countries have shed light on a strong relationship between the richness of the gut microbiota and decreased mortality rates in individuals who have undergone allogeneic HSCT 202. Another comprehensive longitudinal investigation analyzed over 10,000 fecal specimens from patients who underwent allogeneic HSCT and meticulously tracked daily fluctuations in the differential blood cell counts of those patients. The study results implied a close-knit interplay between the dynamics of immune reconstitution and the complex composition of the gut microbiota 203.

This notable connection between the gut microbiota and the various facets of post-transplant biology has been corroborated via experimental findings in murine models, which have further established that the influence of this association extends to nutritional factors, post-transplant bone marrow integrity, thymic cell function, lymphocyte homeostasis, and the intricate processes of hematopoiesis 204. This heterogeneous influence is partly attributable to the endogenous ligands for retinoic acid-inducible gene I, including 3pRNA and RNA from various sources such as viruses, phages, or bacteria. These ligands were found to induce a protective fibronectin I signaling pathway and facilitate intestinal barrier repair in the intestinal cells 205. The generation of lymphocytes in the post-transplant phase is also intrinsically linked to the efficient extraction of energy from dietary sources, which may hinge upon the genomic repertoire of the carbohydrate-active enzymes in the gut microbiota 204.

#### 2.1.3 Role of gut microbiota in TME regulation

The complicated impact of the gut microbiome extends to local and distant tumors, coordinating a complex interplay involving the modulation of immune responses, bone marrow and lymphocyte trafficking, inflammation, and metabolic patterns. In this intricate network, secretions from the gut microbiota are the primary players. For example, commensal microorganisms release outer membrane vesicles that have the remarkable capability to reprogram the TME toward a pro-Th1 profile, leading to the upregulation of cytokines such as CXCL10 and IFN-γ 206. Moreover, metabolites, including butyrate and nicotinic acid, from these microorganisms serve as mediators of G-coupled receptor-109a-dependent induction of IL-18 in the colonic epithelial cells, along with the dampening of colitis and colitis-associated tumorigenesis 207. Additionally, the nicotinamide adenine dinucleotide phosphate oxidase 2-mediated decrease in the production of myeloid cell reactive oxygen species in the context of tumor-associated oxidative stress after antibiotic treatment or in germ-free conditions was reported to compromise the effectiveness of tumor therapies, thus highlighting the cooperative role of commensal microorganisms in cancer development 208. The mono-association of gnotobiotic mice with specific bacteria in colonized gut-draining LNs induces CTLs to produce TNFα 209. Furthermore, endogenous gut bacterial translocation in Tet2-deficient mice driven by Tet2 intrinsic factors was found to be crucial in instigating IL-6-dependent pre-leukemic myeloproliferation. Moreover, this phenomenon was amendable via antibiotic intervention and completely abrogated in germ-free mice, indicating its potential application in clinical management 210,211. However, subsequent findings indicated that a complete gut microbiota was essential in preventing leukemia progression in genetically predisposed mice.

Non-hematopoietic constituents of the gut mucosa also have a crucial association with the TME. Genetic deficiencies in mice and bone marrow chimeras have unveiled the integral role of ring-finger protein 5 (RNF5), an E3 ubiquitin ligase, in melanoma immune surveillance. Specifically, the absence of RNF5 in mice resulted in the diminished secretion of antimicrobial peptides and heightened epithelial cell apoptosis in cryptopatches, ultimately altering the gut microbiota composition. This mucosal damage, in turn, increased DC mobilization toward melanoma-draining LNs, resulting in the enhanced intratumoral infiltration of IFN-γ-generating T lymphocytes. Furthermore, cohousing Rnf5^-/-^ and wild-type mice or administering antibiotics confirmed the microbiota-mediated effects and ultimately restored tumor invasiveness 187. Another study observed a correlation between the overrepresentation of immunogenic bacteria (particularly TLR2 agonists) and oxaliplatin-induced crypt cell apoptosis in the ileal mucosa 184. This finding corresponded to the priming of TFH cells in LNs, ultimately leading to B cell activation, Ig production, and TIL infiltration in patients and mice with colon cancer. Moreover, the disruption of the intestinal barrier function by anti-CTLA4 treatment was found to be vital for the systemic translocation of adenosine derived from *Bifidobacterium*, consequently promoting Th1 activation and anti-tumor immunity via T cell-specific A2AR signaling 212. All these results strongly suggest that gut barrier dysfunction or the translocation of microbiota metabolites is closely tied to the composition of local microbial communities, which in turn mobilizes DCs within and outside GALTs and significantly contributes to T-cell infiltration in the TME.

Additionally, the immune components within tumors not only encompass stromal, tumor, and endothelial cells and hematopoietic progenitors but also comprise a compact network of intricate connections to adrenergic nerve fibers 213-216. Further, the neuron subsets within the gut nervous system are responsive to the gut microbiota and function in a region-dependent manner to independently influence metabolic control beyond the regulation by the central nervous system 217. All these findings imply a close relationship between mucosal or tumor-associated commensal microorganisms and the nerve fibers innervating the tumors, thereby warranting further exploration in this aspect.

### 2.2 Microbiota at extraintestinal barriers and its influence on cancer immunity

Considering that the intestinal barrier is the most expansive interface between the host organism and its microbiota and has the highest microbial diversity, research endeavors have predominantly investigated the impact of the gut microbiota on cancer development and prognosis 179. These investigations can potentially uncover the causal relationships between alterations in gut microbiota composition and the impairment of tumor immunosurveillance. More importantly, these effects may extend beyond intestinal malignancies and include extraintestinal cancers. However, extraintestinal cancers have often been shown to originate in tissues harboring their distinct microbiota, indicating that these microbial ecosystems may be pivotal in tumor progression 218-220.

For example, the lung, which has a substantial surface area of approximately 1 m^2^/kg of body weight, does harbor microorganisms 221. In the case of oncogene-driven native lung cancer, studies involving murine models have suggested that local symbiotic relationships may be disrupted during carcinogenesis. These changes initiate complex interactions between alveolar macrophages and lung-residing γδ T cells, ultimately resulting in lung tumor advancement 222. In line with this finding, Le Noci *et al.* demonstrated that the reduction in bacterial biomass owing to the administration of aerosolized antibiotics was associated with enhanced anti-tumor immune responses, possibly involving the activation of T and NK cells, as well as a decrease in immunosuppressive Tregs. Furthermore, the use of probiotic *Lactobacillus rhamnosus* GG could mitigate immunosuppression, inhibit lung tumor engraftment, and decrease tumor metastasis under antibiotic and probiotic conditions 223. The clinical relevance of these observations has recently become prominent in a study involving patients with lung cancer 224. In that study, Tsay *et al.* reported that the microaspiration of upper airway symbionts in patients with lung cancer substantially influences therapy response and overall survival (OS). This result may be closely linked to the aggravation of Th17-mediated inflammation, an expected consequence of immune checkpoint inhibition 224. Another research by Greathouse *et al.* has proposed a connection between TP53 and changes in the lung microbiota. In particular, they detected an abundance of the *Acidovorax* genus in lung biopsy specimens from individuals with squamous cell carcinoma, along with the further enrichment of a comparable taxon in lung biopsies of patients with squamous cell carcinoma and TP53 mutations 225.

The skin, which is the largest and outermost organ of the human body, plays a pivotal role in preserving host homeostasis by actively communicating with its resident microbiota, keratinocytes, and an array of skin immune components 221. This intricate interplay occurs through a nexus of metabolic, innate, and homologous immune responses. Previous studies have demonstrated that alterations in the skin microbiota composition can significantly affect the onset of non-melanoma skin carcinogenesis 226. In support of this notion, a cell culture study incorporating *Staphylococcus epidermidis*, a specific strain of skin commensal bacterium, exhibited a potent protective effect against skin cancer, emphasizing the profound impact of the commensal microorganisms on the skin. These *S. epidermidis* strains were found to produce 6-N-hydroxyaminopurine, a DNA polymerase activity inhibitor capable of suppressing the proliferation of tumor cell lines in culture 227. Hoste *et al.* employed a mouse model of wound-induced skin cancer to delve deeper into the mechanisms underlying the promotion of inflammation and tumorigenesis by skin microbiota. Their investigation revealed that skin microbiota was indispensable for promoting inflammation and tumorigenesis. Furthermore, eliminating skin microbiota prevented tumor development, primarily by dismantling several key innate immune sensors, including TLR5, with inflammation as a pivotal correlate of tumorigenesis. Lastly, they observed that antibiotic treatment effectively inhibited tumor formation in a TLR5-dependent manner 228.

Correspondingly, cervical cancers arising from persistent high-risk human papillomavirus infections are frequently linked to an imbalance in cervical microbial communities 229,230. These intricate interactions emerging between microbial symbionts and virus-related cancers, along with their potential synergistic effects on tumorigenesis, necessitate thorough investigation. Therefore, the cancer-microbiota interactions at extraintestinal barriers beyond the gut barrier should be comprehensively explored.

### 2.3 Impact of intratumoral microbiota on the TME

**Figure 4:**
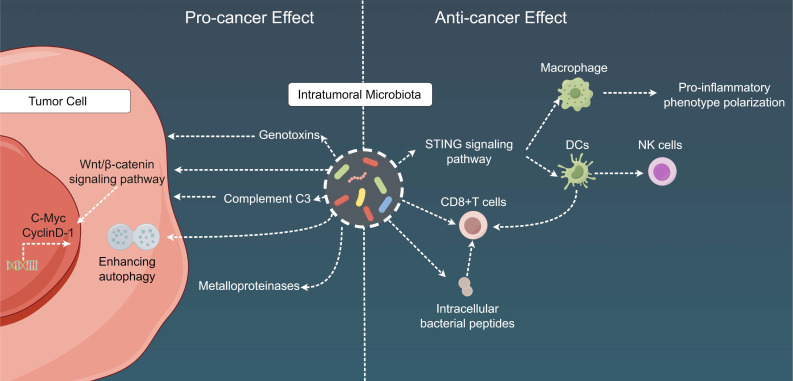
** Mechanistic insights into the dual role of intratumoral microbiota in cancer.** Intratumoral microbiota play a dual role in the TME. On the one hand, intra-tumoral bacteria contribute to tumorigenesis, progression, drug resistance, and metastasis by releasing genotoxins, promoting the WNT/β-catenin signaling pathway, inducing host complement C3, fostering cancer cell autophagy, and secreting metalloproteinases. On the other hand, these intra-tumoral bacteria facilitate anti-tumor immunity through mechanisms involving immune cell recruitment, activation of CD8+ T cells, induction of chemotactic factors, and stimulation of anti-tumor immune responses via bacterial antigen elicitation. TME: Tumor microenvironment; DC: Dendritic cell; STING: Stimulator of interferon genes.

In-depth studies on the mechanistic underpinnings of intratumoral microbiota are notably scarce. Nevertheless, previous investigations have demonstrated the multifaceted effects of this microbiota on the TME, highlighting their potential complex modulations of local anti-tumor immunity 231-233 (Fig. 4). Current research indicates that the intratumoral microbial community predominantly colonizes within tumors through three main routes. The first route involves mucosal barrier origins, encompassing gastrointestinal tumors such as colorectal and pancreatic cancers, as well as pulmonary and cervical cancers. Given that these organs possess externalized cavities, the microbes colonizing the mucosal surfaces may infiltrate tumors via mucosal disruptions occurring during tumorigenesis 231,234. The second route is via neighboring normal tissues. Prior studies have detected certain bacteria within organs that were initially considered sterile, with the microbial composition in the tumor tissue closely resembling that in the adjacent normal cellular tissue. Furthermore, the immunosuppressive milieu and hypoxic microenvironment of tumors have been found to facilitate microbial colonization 177. However, the source of microbiota in normal tissues remains unclear and may have spread from tumor sites; thus, additional research is required to substantiate this notion. Finally, the third possible route of intratumoral microbial colonization is through hematogenous dissemination, wherein microbiota originating from the oral cavity, intestines, and other potential sites may be transported to tumor locations via the bloodstream and gain entry into tumors via compromised vascular permeation 235.

Additionally, intratumoral microbiota can exert the following cancer-specific effects: (1) influence carcinogenesis in the gastrointestinal and urogenital tracts, mainly through the secretion of genotoxins, such as colibactin toxin from pks^+^ *Escherichia coli* and toxins from *B. fragilis*^236^-^239^; (2) affect chemotherapy resistance either directly via microbial metabolism (e.g., gemcitabine degradation by cytidine deaminase in pancreatic cancer) or indirectly by augmenting cancer cell autophagy in colon cancer^240^,^241^; (3) promote tumor proliferation in pancreatic cancer through the fungal activation of the host C3 complement cascade^242^; (4) escalate breast and lung cancer metastasis via the upregulation of tumor stromal metalloproteinases or the attenuation of tumor immunosurveillance^243^,^244^; and (5) engage with host oncogenic pathways through microbial products within the TME, potentially resulting in the upregulation or activation of these oncogenic pathways, with a particular impact on specific pathways such as the Wnt/β-catenin signaling pathway. Disruptions in β-catenin signal transduction can stimulate the transcription of key cancer-associated genes, including cellular myelocytomatosis oncogene and cyclin D-1, thereby contributing to the advancement of carcinogenesis and tumor progression 245,246. In terms of immunological ramifications, intratumoral microbial populations frequently establish tolerogenic programming via their interactions with pattern recognition receptors, which can result in diminished proportions of TILs (such as CD8 T cells) and an increased number of Tregs. These findings have been documented in lung, breast, colorectal, and pancreatic cancers 243,244,247-250. Moreover, multiple studies have implied that imbalances within local bacterial communities can elicit a persistent proinflammatory immune response, thus fostering cancer progression. For instance, this phenomenon may develop from the microbial activation of NF-κB, a pivotal regulatory factor in cancer-associated inflammation 246,251,252.

Recent research has also determined that tumor-related microbiota can bolster anti-tumor immunity through multiple mechanisms. For example, bacteria, such as *Bifidobacterium*, migrate to colorectal cancer (CRC) sites, where they establish residence and subsequently activate DCs via the stimulator of interferon genes (STING) signaling pathway 186. Additionally, STING agonists derived from *Akkermansia muciniphila* have been shown to stimulate IFN-I production by intra-tumoral monocytes, thus promoting macrophage reprogramming and communication between NK cells and DCs. Ultimately, these changes were found to enhance the effectiveness of ICI in patients with melanoma 253. Another mechanism by which the tumor microbiome can shape anti-tumor immunity is encouraging the recruitment and activation of CD8^+^ T cells. For example, *Saccharopolyspora*,* Pseudoxanthomonas*, and *Streptomyces* in pancreatic ductal adenocarcinoma (PDAC) tissues were demonstrated to favor the activation of CD8 T cells, resulting in anti-tumor immune responses 254. Furthermore, the tumor microbiome, including lactobacilli, Epstein-Barr virus, hepatitis B virus, and Merkel cell polyomavirus, can induce chemokine production, thereby influencing the infiltration of CD8 T cells into tumor tissues and potentially enhancing the survival rates of patients with cutaneous melanoma 255-259. Lastly, antigens originating from the tumor-associated microbiome can also provoke anti-tumor immune responses. One study revealed an increase in IFN-γ-secreting and melanoma-infiltrating lymphocytes after exposure to various bacterial peptides compared to control cells not loaded with these peptides. This finding indicates that intracellular bacterial peptides presented by tumor cells can trigger T-cell immune responses, possibly serving as viable targets for combating tumor cells 260. Furthermore, other studies have revealed that the intratumoral administration of bacteria or their antigens can paradoxically induce immune stimulatory effects, as observed in notable research based on Coley's toxins and bacterial cancer therapies 19,261. Experiments involving intratumoral *Bacteroides* in the context of breast cancer have underscored the significance of lymphoid lineage cells as intermediaries influencing the impact of intratumoral microbial communities on tumor immune surveillance 243. However, the precise mechanistic basis of these associations remains unknown.

## 3 Impact of microbiota-associated therapy and antibiotics on cancer immunotherapy

### 3.1 Impact of microbiota on cancer immunotherapy response

Mounting evidence suggests that microbiota members could serve as prognostic biomarkers for predicting patient responses to immune checkpoint blockade (ICB) therapy. Previous researchers have identified substantial differences in the microbiome composition of patients with varying ICB prognoses (Table 2) 180,262. For instance, a pioneering global prospective study analyzed the fecal samples of 39 patients with melanoma who underwent combined immunotherapy with anti-CTLA4 (ipilimumab) and anti-PD-1 (nivolumab) or anti-PD-1 treatment alone 263. The results showed that *Holdemania filiformis*, *B. thetaiotaomicron*, and *F. prausnitzii* were enriched in patients responding favorably to the combination therapy, whereas *Dorea formicigenerans* was abundant in those responding to anti-PD-1 treatment 263. Additionally, 16S ribosomal RNA sequencing of archived fecal samples unveiled increased proportions of *Burkholderiales* and *Bacteroidales* in the anti-CTLA4 responders, highlighting the role of CTLA4 blockade-induced mucosal damage in regulating the microbiota 169. In the case of non-melanoma cancers, a relatively larger cohort of patients with advanced epithelial tumors demonstrated a correlation between a higher abundance of *muciniphila* bacteria in their feces and more favorable responses to anti-PD-1 therapy 170. Moreover, FMT from responders (R-FMT) and non-responders (NR-FMT) to germ-free mice provided compelling evidence for the modulatory role of microbiota in anti-tumor immune responses 169,170. The NR-FMT mice displayed enhanced tumor growth rates and diminished responsiveness to anti-PD-1 therapy compared to their R-FMT counterparts, thereby reinforcing the notion that microbiota has a crucial regulatory function in ICI therapy.

Although the link between the microbiota and ICB therapy response is evident, the exact fundamental mechanisms are still ambiguous. Most researchers have concentrated on the adaptive immune responses influenced by the microbiota during ICB therapy. One plausible mechanism posits that the microbiota augments CD8 T-cell responses against tumors, with *Bacteroides* abundance being linked to CD8 T-cell activity in early mouse experiments. Furthermore, a probiotic mixture containing live *Bifidobacterium* spp*.* was found to inhibit melanoma growth and enhance tumor-specific CD8 T-cell responses, similar to that observed in anti-PD-L1 therapy 168. In a clinical trial of patients with NSCLC, responders showed increased levels of the *Enterococcus hirae* strain 13144. These increased proportions of *E. hirae* corresponded to heightened peripheral CD8 and CD4 T-cell responses, amplified IFN-γ production, and an extended PFS 170. Another study of healthy donor feces identified 11 bacterial strains capable of promoting the accumulation and recruitment of intestinal IFN-γ CD103 T cells. This ability of the bacterial strains was independent of innate immune regulation and instead relied on resident lamina propria DCs and MHC Ia class molecules 194.

An additional potential mechanism pertains to the impact of microbiota on Th1 immune responses and its subsequent influence on the TME. The immunogenicity of certain *Bacteroides* or *Thauomyces* spp. has been associated with IL-12-dependent Th1 immune responses, which in turn have implications for the effectiveness of anti-CTLA4 therapy 169. In a rearranged during transfection (RET) melanoma mouse model, a 2-week broad-spectrum antibiotic regimen in germ-free and specific pathogen-free mice led to reduced anti-CTLA4 effects. However, orally supplementing the mice with a mixture of *B. fragilis* and *Burkholderia cepacia* resulted in enhanced LN and intratumoral DC maturation, culminating in heightened Th1 immune responses and restored anti-CTLA4 efficacy 169.

Microbiota may also affect the TME via its influence on Th17 cells. Earlier investigations have explored whether metastatic PDAC harbors a microbiota composition similar to that of the gut. After oral antibiotic administration, the unique TME of PDAC, characterized by IL-17A CD4 Th17 cells that inhibit the differentiation of anti-cancer IFN-γ CD4 Th1 cells, exhibited a significant reduction in tumor burden and a shift toward immunogenicity, with the shift being particularly evident in the context of adaptive immune responses 248.

Moreover, *A. muciniphila* has been identified as a microbiota member influencing ICB responses through innate immunity modulation. Although the specific mechanisms are yet to be deciphered, *Akkermansia* has emerged as a possible candidate for predicting or enhancing ICB responses owing to its reported mucosal healing capabilities. For example, the oral administration of *Akkermansia* in NR-FMT mice preserved the efficacy of anti-PD-1 therapy, with IL-12 promoting the recruitment of CCR9^+^CXCR3^+^CD4^+^T lymphocytes to the tumor bed 264. Another study found that the concurrent administration of *Akkermansia* and *Prevotella copri* in germ-free mice significantly potentiated anti-PD-1 therapy effectiveness against metastatic melanoma, NSCLC, and RCC 170. Furthermore, a previous investigation highlighted the role of *Akkermansia* in stimulating T follicular helper cell-dependent IgG1 responses in mice. However, the precise processes underlying these effects of *Akkermansia* require further elucidation, with its association with type 1 immunity being proposed as a potential mechanism 265.

Initial evidence on the association between microbiota and ACT effectiveness was observed in a murine model with a deficiency in CD14 and TLR4 receptors 167. This association manifested after combining ACT with total body irradiation (TBI), a form of lymphodepletion. The modulation of the microbiota via antibiotic treatment or inhibiting lipopolysaccharide (LPS) signaling constituents ultimately resulted in the impaired functionality of the infused CD8^+^ T cells and a decrease in activated DCs. Consequently, the therapeutic efficacy of ACT was compromised. In contrast, introducing LPS to TBI-treated, microbiota-depleted mice substantially amplified the proliferation and functionality of reinfused T cells and even induced sustained remission in mice with sizable tumors. In terms of the underlying mechanism, the TBI procedure is suggested to trigger microbial translocation, particularly that of gram-negative bacteria proficient in LPS production, into the mesenteric LNs. These translocated microorganisms, in turn, activate the TLR4 pathway by expressing varied TLR4 agonists, including LPS and peptidoglycan. This alteration subsequently heightens DC activation and increases the secretion of pro-inflammatory cytokines, including IL-1β, IL-6, and TNF-α, within the gastrointestinal tract. LPS administration has also been found to bolster the anti-cancer response mediated by the transferred CD8^+^ T cells in TBI-untreated mice 167.

Uribe-Herranz *et al.* investigated the administration of ACT for treating tumors in C57BL/6 mice procured from two distinct sources: the Jackson Laboratory (JAX) and Harvard (HAR) 266. The results unveiled a marked contrast in the tumor growth between these two sets of mice. The HAR mice exhibited nearly complete suppression of tumor growth, whereas the JAX mice did not show any such response. Subsequent analysis of 16S rRNA sequencing data demonstrated obvious differences in the fecal microbiota composition between these two mice cohorts. The HAR mice displayed a diverse array of *Bacteroidetes* taxa, while the JAX mice were predominantly characterized by *Bacteroidales S24-7*. This difference hinted at a potential correlation between ACT efficacy and specific genera of *Bacteroidetes*, specifically *Bacteroides* and *Parabacteroides*. Subsequently, the JAX and HAR mice were administered vancomycin, an antibiotic targeting the gram-negative *Bacteroidetes* phylum, to further assess this correlation. The vancomycin intervention had no significant effect on the HAR mice, whereas it remarkably enhanced tumor regression in the JAX mice, effectively aligning their response with that of the HAR mice without vancomycin treatment. This improved response in the JAX mice was ascribed to a bolstered Th-1-mediated immune response and an increased accumulation of peripheral DCs, all of which led to the heightened expansion and activity of the transferred T cells 266. Moreover, the antibiotic treatments employing neomycin and metronidazole failed to induce any discernible phenotypic changes, highlighting the specific role of certain bacterial species in orchestrating the host response to ACT.

However, the constrained approval timeline has caused a scarcity of available data on the association of ACT effectiveness and tolerance with the microbiota. Hence, controlled investigations are imperative to evaluate the fecal bacterial communities in these patient cohorts. The diversity of prior treatments and sample profile variability also pose significant challenges to attaining satisfactory outcomes. Nevertheless, the current recommendation still advocates broad-spectrum antibiotics for patients undergoing CAR-T cell therapy and autologous transplantation 267.

### 3.2 Impact of microbiota transplantation and dietary regulation on cancer immunotherapy efficacy

FMT has been granted FDA approval for treating recurrent and refractory *Clostridioides difficile* infections 268. Preliminary research by Wang *et al.* has additionally highlighted the transformative potential of FMT in immunotherapy by detailing the successful treatment of immunotherapy-induced colitis using FMT enriched with beneficial *Bifidobacterium* species 269. However, this initial study warrants future clinical trials to validate the therapeutic potential of FMT in immunotherapy. Moreover, recent proof-of-concept clinical trials have established the safety and efficacy of FMT in augmenting anti-PD-1 monoclonal antibody responses in patients with refractory melanoma. These studies indicated that combining microbial depletion via antibiotic treatment with subsequent FMT and reinitiation of anti-PD-1 monoclonal antibody treatment resulted in partial or complete remission in select patients. This outcome underscores the therapeutic capability of FMT in strengthening immunotherapy responses, with its characteristic sustained impact on the gut microbiota and the requirement for fewer frequent interventions than other modulation methods 8. Currently, a series of clinical trials investigating the use of microbiota transplantation in boosting immunotherapy efficacy is underway (Table 3). Nonetheless, future research undertakings should incorporate ICI biomarkers, including PD-L1 expression and tumor mutational burden, to assess the indispensable role of pre-existing adaptive immunity in facilitating effective FMT 270,271.

Another approach to enhance ICI responses involves the application of prebiotics and dietary interventions. Common prebiotics, including inulin and oligofructose, are capable of enriching beneficial bacterial species such as *Bifidobacterium*, *Lactobacillus*, and *Faecalibacterium* in the human gut 272,273, yielding improved anti-cancer immune responses 168,169,194. However, additional research is essential to elucidate the direct impact of prebiotics on ICIs. Dietary modulation has also exhibited efficacy in improving treatment outcomes. For instance, high-fiber diets have been associated with the enrichment of *Bifidobacterium* species and improved clinical outcomes in patients with metastatic NSCLC and melanoma undergoing ICI 274. Mechanistic studies support these findings, illustrating that high-fiber diets elevate TIL levels in ICI-treated mice and that fiber supplementation leads to the enrichment of the *Ruminococcaceae* family that in turn facilitates T-cell activation and tumor infiltration 7. Additionally, ketogenic diets and the resulting ketone bodies have proven instrumental in augmenting ICI efficacy in mice by promoting CD8^+^ T-cell proliferation and suppressing PD-L1 expression, thereby sustaining T-cell activation to exert anti-cancer effects 275. Furthermore, the rapid responses of microbiota to dietary changes are noteworthy and suggest the transient nature of dietary modulation 276,277. Therefore, comprehensive research should focus on strategies to prolong and sustain dietary effects for achieving optimal ICI responses.

A parallel strategy to modulate immunotherapeutic responses entails the utilization of probiotics. Commercial probiotics containing microbiota species, such as *B. longum* and *L. rhamnosus* GG, linked to enhanced immunotherapy responses have shown promise in preclinical investigations 168,278. These probiotics have demonstrated potential in enhancing anti-cancer immunity by reducing Treg levels, promoting CD8^+^ T-cell activation and CD4^+^ T-cell differentiation, and facilitating intratumoral NK cell infiltration 279,280. Moreover, a meticulously curated consortium of 11 bacterial strains, comprising seven *Bacteroides* and four non-*Bacteroides* species, has been reported to induce IFN-γ-producing CD8^+^ T cells mediated by CD103^+^ DCs, thereby enhancing ICI efficacy in syngeneic tumor-bearing mice. All these findings stress the potential of probiotics as supplementary agents to amplify immunotherapy effectiveness. Previous clinical trials have observed that certain probiotic strains, especially *Bifidobacterium lactis* Bl-04 and *Lactobacillus acidophilus* NCFM, cause an increase in butyrate-producing species, including *Faecalibacterium* and *Clostridiales,* within the microbiota of patients with CRC. This proliferation coincides with the improved immunotherapy responses 281.

However, current clinical research primarily addresses the impact of probiotics on microbiota composition and does not identify any direct causative links with immunotherapy outcomes. Additionally, recent findings suggest that individuals using probiotics may experience a potential reduction in microbial diversity, a characteristic commonly associated with non-responsiveness to immunotherapy 282. This phenomenon has been substantiated by preclinical mechanistic studies that have shown that mice treated with probiotics including *B. longum* or *L. rhamnosus* GG displayed attenuated responses to anti-PD-L1 monoclonal antibody treatment and reduced levels of IFN-γ^+^ CD8^+^ T cells within the TME, which contrasted prior research findings 7. Consequently, the indiscriminate use of over-the-counter probiotics in patients undergoing immunotherapy should be discouraged due to the existing knowledge gaps.

### 3.3 Impact of antibiotics on cancer immunotherapy efficacy

Antibiotics are a standard intervention in the prophylactic and therapeutic care of patients with cancer, owing to their infection susceptibility. However, recent research suggests that the timing and duration of antibiotic use may significantly influence immunotherapy effectiveness 170,175,283-291 (Table 4). Thus, clinical practitioners must exercise caution when considering antibiotic application in this patient population.

Broad-spectrum antibiotics, even when administered for extended periods in cases of evident or latent infections, have been implicated in disrupting the delicate microbiota balance and impairing immune cell responses 292,293. Moreover, patients who received antibiotics immediately before or after anti-PD-1 treatment experienced an approximately 50% reduction in median survival compared to those not administered antibiotics 170. Similarly, patients with late-stage cancer concurrently using antibiotics with ICI therapy demonstrated diminished response rates and shorter OS or PFS 288,294. Prolonged antibiotic exposure is also positively associated with the risk of various cancers 284,285,295-297. In line with these results, murine models of NSCLC and melanoma revealed that the co-administration of broad-spectrum antibiotics, such as vancomycin, ampicillin, metronidazole, and neomycin, suppressed the protective IL-17-producing γδT17 cell response, ultimately promoting metastasis 298. All these findings underscore the adverse effects of antibiotics on tumor progression.

Nonetheless, the existing body of evidence on antibiotic use and cancer treatments primarily consists of animal experiments and retrospective investigations, making it challenging to confirm the direct negative impact of antibiotics on anti-PD-1 efficacy. Hence, prospective research is necessary to validate these research results. A multicenter, prospective cohort study involving 196 patients with NSCLC, melanoma, RCC, or head and neck cancer reported that those who received antibiotics prior to PD-1/PD-L1 antibody treatment had poorer responses and OS 299. Furthermore, patients who were administered a single dose of broad-spectrum antibiotics 1 month before ICB treatment fared worse clinically than those undergoing antibiotic therapy simultaneously with ICB treatment 299. Additionally, ICB treatment following antibiotic administration appeared less favorable than no initial treatment. Another clinical trial assessed the outcomes of patients with late-stage RCC or NSCLC who received anti-PD-L1 monoclonal antibodies alone or in combination with antibiotics (quinolones or β-lactams) within 4 days of treatment initiation and observation that the addition of antibiotics reduced OS in NSCLC and PFS in those with RCC 291. The study also found that patients receiving antibiotics within the first 30 days before ICB treatment experienced poorer clinical outcomes than those receiving antibiotics within the first 60 days 291. Overall, these findings emphasize the critical significance of antibiotic administration timing in immunotherapy.

Moreover, specific antibiotics, including ampicillin, vancomycin, and lincomycin as well as imipenem, are linked with microbiota alterations, which in turn affect anti-CTLA4 therapy and weaken anti-tumor effects 169. Studies involving K/BxN mice have consistently proven the potent inhibitory effects of antibiotics, such as vancomycin and ampicillin, on the progression of rheumatoid arthritis. However, a broader microbiota-directed antibiotic regimen incorporating vancomycin to target gram-negative bacteria, metronidazole and neomycin for anaerobic bacteria, and ampicillin for gram-positive bacteria led to a reduction in Th17 cell populations, with each antibiotic acting via distinct mechanisms 300,301. Conversely, microbiota restoration was able to achieve Th17 cell recovery, suggesting that microbiota regulation can mitigate antibiotic-induced immune dysfunction and influence the occurrence and severity of gut lesions caused by antibiotic therapy, ultimately enhancing therapeutic efficacy.

Some researchers have reported contrasting findings, wherein antibiotics were synergistically utilized with other drugs to elicit anti-cancer effects. For example, a lipid delivery system co-loading curcumin and doxorubicin has been developed to exploit their synergistic anti-cancer effects, resulting in tumor attenuated and efficient therapeutic outcomes and consequently enhancing tumor control 302. The antibiotic tigecycline has emerged as an effective agent for highly malignant double-hit lymphoma, with characteristic activation of MYC and B-cell lymphoma-2 cancer genes. The combination of tigecycline with B-cell lymphoma-2 inhibitors such as venetoclax exhibited significant anti-cancer effects, presenting a promising front-line treatment strategy for lymphomas 303. The addition of antibiotics offers advantages in modulating the effects of antibiotic-induced imbalance of a single microbial community 304. Given the propensity for gut tumors to co-occur with microbial dysbiosis, polysaccharide regulation may be a potent approach to maintaining a balanced gut microbiota, with studies revealing its ability to counteract gut microbiota dysbiosis caused by paclitaxel chemotherapy in a 4T1 breast cancer mouse model 305.

Furthermore, combining antibiotics with radiation therapy has yielded improved treatment outcomes. For example, the addition of vancomycin to radiation therapy was superior to individual drug utilization in model animals, leading to modification of the TME and enhancement of local antigen presentation, as well as tumor infiltration by IFN-γ- and CD8-dependent cytotoxic T cells 306. A positive correlation has also been reported between antibiotic use and tumor immunogenicity. For instance, erythromycin, a clinically effective macrolide-type anti-tumor antibiotic, was found to enhance tumor-infiltrating T-cell populations. *Fusobacterium nucleatum* is commonly observed in CRC tissues, and metronidazole treatment of colon cancer xenograft mice was shown to reduce these bacterial loads in the TME and slow tumor cell proliferation 185. Additionally, short-chain fatty acids (SCFAs) generated by the gut microbiota, particularly clostridia, exert inhibitory effects on APC function and diminish the radiotherapy-boosted anti-tumor efficacy of vancomycin. Moreover, vancomycin has been demonstrated to specifically modulate gram-positive bacterial populations, such as clostridia, leading to reduced SCFA and C4 concentrations within fecal and tissue samples. These findings underscore the benefits of precision antibiotic therapy aimed at cancer-associated microbial communities, thereby laying the groundwork for the development of potential therapeutic strategies for treating individuals with cancer 185. Antibiotic interventions in the gut microbiota positively affect tumor immunity. In some patients with pancreatic cancer, the gut microbiota was found to promote immunosuppression and Treg cell proliferation via Foxp3 induction 307. A judicious application of antibiotics can be used to regulate the advancement of premalignant lesions and cancer progression. Butyrate has been shown to induce alterations in T-cell behavior via epigenetic modifications in the Foxp3 gene, indicating that antibiotics can influence the gut microbiota to alter the generation of these critical metabolic byproducts. Antibiotic cocktails (ABX) can disrupt microbiota, ultimately suppressing PDAC infiltration and inducing immunogenic reprogramming of the TME after its dissolution. ABX administration is also associated with increased M1 macrophage and Th1 CD4^+^ T-cell differentiation and CD8^+^ T-cell activation, as well as significantly upregulates PD-1 expression on effector T cells and promotes checkpoint-targeted immunotherapy outcomes. All these results imply that antibiotics can assist in enhancing cancer immunotherapy by establishing an equilibrium within microbial communities, influencing tumor-associated microbiota, and augmenting anti-tumor advantages 9. Therefore, formulating oral antibiotics to target the microbiota is a promising approach for further improving immunotherapy outcomes.

In light of these findings, the use of antibiotics and probiotics to restore a healthy gut microenvironment is especially relevant. Correspondingly, previous studies have observed that replenishing the gut microbiota in mice effectively reversed the intratumoral immunogenic alterations in their tumors, which were initially ascribed to the depletion of bacteria by antibiotic treatment. This reversal also translated into the increased expression of genes linked to T cell-mediated immune activation in the tumors of mice that had received antibiotic treatment. These results indicated that the reconstituted microbiota could more effectively regulate immune activation. Furthermore, antibiotics aid in preventing gut bacterial translocation, thus maintaining a stable distribution of bacteria in the body. For example, ABX administration efficiently mitigated bacterial translocation to the liver and gut, wherein the disruption of the gut vascular barrier was responsible for the systemic dissemination of gut bacteria and the subsequent colonization of the liver in CRC 248.

As mentioned above, prior studies have unveiled a compelling association between antibiotic utilization and lower PFS, OS, and response rates, with the timing of antibiotic administration being critical. A comprehensive meta-analysis has noted that patients without antibiotic exposure within 42 days before commencing ICI therapy had 3.43 times longer OS than those who received antibiotics within 60 days preceding ICI initiation 308. These findings are congruent with research reporting the restoration of microbial composition to nearly baseline levels within 42 days after administering healthy individuals with an ABX (meropenem, gentamicin, and vancomycin) for 4 days 309.

Apart from epidemiological observations, emerging studies have delineated distinct microbial signatures in antibiotics-exposed patients, with a characteristic reduction in microbial diversity and enrichment of *Clostridium hathewayi* and features associated with diminished survival rates. Consequently, a prudent approach should be followed, wherein antibiotic use is avoided before initiating ICI therapy 175,290. Alternatively, FMT or probiotics may be a viable option to rectify antibiotic-induced dysbiosis preceding ICI therapy. However, treating PDAC may require a different strategy because preclinical study findings indicate that antibiotics targeting intratumoral bacteria can potentiate immunotherapy efficacy in PDAC 248.

Based on all these results, although antibiotic use may hinder anti-cancer responses to some extent by influencing the microbial composition, the potential to expand the richness and diversity of the gut microbiota via the synergistic interactions between different antibiotic drugs should not be underestimated. Additionally, meticulously assessing the influence of antibiotics and microbiota across diverse cancer types is imperative, considering the varied factors including host immune status, tumor genetic factors, TME, and microbiota modulation. Lastly, remarkable improvements in tumor control can be achieved by developing antibiotic combinations to suppress tumor development, regulating the antibiotic-induced ecological imbalance that can lead to tumor progression, and enhancing the efficacy of antibiotic detoxification within the TME.

## 4. Conclusion and prospects

Previous studies have made substantial progress in comprehending the role of the host microbiota in normal physiology and disease, as well as shed light on the potential therapeutic strategy of targeting microbial communities within the gastrointestinal tract and other ecological niches for treating diseases and promoting holistic well-being. However, this field is still at a nascent stage, displaying valuable perspectives for further unraveling the mechanisms by which these microorganisms affect various physiological and pathological processes. Moreover, identifying the most effective approach, such as dietary interventions and other modalities, for targeting these microorganisms is vital.

Further, the increasing recognition of the microbiome as a pivotal determinant of health and disease has raised the prospect of incorporating the assessment and regulation of microbial communities in the gut and other ecological habitats into precision oncology care. This convergent application can potentially foster the evolution of comprehensive precision health paradigms. Currently, personalized cancer care involves the histopathological characterization of precancerous or cancerous tissues via targeted gene profiling or next-generation sequencing methods 310,311. Other processes employed include the analysis of genomic or proteomic alterations, as well as the limited assessment of immune cells (utilizing PD-L1, CD8, and other biomarkers) at baseline and during treatment to guide therapy and determine treatment responses 312-314.

Recent developments have introduced more comprehensive strategies for cancer prevention and treatment 195,277, with the potential to improve health using multifaceted monitoring, feedback, and early interventions. These advances enable a more holistic approach to cancer care and involve the assessment of somatic and lineage mutations in tissue/tumors and blood, as well as the feature evaluation of microbiota derived from tissue and blood samples. Thus, in-depth examinations of systemic and tissue/tumor-based immunity beyond the current capabilities of conventional biomarkers are warranted. Such assessments will provide a foundation to explore innovative immune mechanisms and can revolutionize cancer prevention and treatment approaches by enhancing immune surveillance 315.

The analysis of microbial communities in the gastrointestinal tract and other ecological niches has indeed shown promise in the field of oncology, particularly in understanding the complex interplay between the host immune system and cancer development. In addition to investigating systemic inflammation markers and lifestyle factors 316, recent research focused on the role of immune-modulating metabolites or compounds within the microbiome that could potentially influence cancer progression and treatment response 317. Immunostimulatory microbial metabolites, such as SCFAs, polysaccharides, and LPS, have been shown to modulate the immune response of host cells 195. Moreover, the manipulation of the microbiome through methods such as FMT or the use of narrow-spectrum antibiotics can be tailored based on individual patient data monitoring. By identifying specific microbial signatures associated with favorable or adverse cancer outcomes, healthcare providers can design personalized dietary interventions and microbial community manipulations to modulate the levels of these immunostimulatory metabolites. Fusing multi-omics data using artificial intelligence also facilitates the implementation of mathematical modeling and other methodologies in this field, thus bolstering strategies for the treatment, interception, and prevention of cancer. This process entails a dynamic cycle of iteration and refinement of current techniques 318,319.

However, current research in this budding field is accompanied by certain limitations. The complexity of the microbiome and its interaction with the host immune system poses challenges in differentiating causation from correlation. Moreover, identifying optimal strategies for manipulating microbial communities continues to be a hurdle due to the variations in individual responses to interventions. Additionally, ethical considerations, including the long-term effects of the interventions and potential unintended consequences, require careful examination.

Furthermore, the possible impact of microbiome-focused strategies on tumor immunotherapy necessitates thorough investigation. Although these strategies may be valuable in enhancing immune surveillance, the chances for unforeseen outcomes, such as exacerbating inflammation or compromising the efficacy of existing immunotherapies, must be carefully evaluated. Thus, the future direction not only involves expanding our understanding of the microbiome but also encompasses critically assessing the translational implications of such interventions in cancer immunotherapy.

In conclusion, integrating microbiome research into precision oncology may be a promising avenue with transformative potential. However, these developments should be considered with caution and a clear recognition of the existing limitations. Thus, future research addressing these challenges can help pave the way for innovative and personalized approaches to cancer prevention and treatment, thereby providing profound implications for the field of tumor immunotherapy.

## Figures and Tables

**Table 1:** The intricate relationship between gut microbiota and tumors.

Gut Microbiota	Model	Mechanism
*Enterococcus hirae;* *Bacteroides intestinihominis*	C57BL / 6J mice, MCA205 sarcoma cell, Ret melanoma cell	*Enterococcus hirae* increased the intratumoral CD8/Treg ratio and *Bacteroides intestinihominis* promoted the infiltration of IFN-γ-producing γδT cells in cancer lesions 183.
*Bacteroides thetaiotaomicron;* *Bacteroides fragilis*	C57BL/6J and BALB/c mice, mouse fibrosarcoma MCA205 cell, murine colon carcinoma MC38 cell, RET melanoma cell, mouse colon carcinoma CT26 cell	The efficacy of CTLA4 blockade is influenced by the microbiota composition. The microbiota composition affects IL-12-dependent TH1 immune responses 169.
*Bifidobacterium spp.*	C57BL/6 mice, melanoma cell line B16 F10, MB49 bladder cancer cell	Commensal Bifidobacterium-derived signals modulated the activation of DCs in the steady state, which in turn supports improved effector function of tumor-specific CD8+ T cells 168.
*Bifidobacterium breve*	C57BL/6 mice, 2C TCR transgenic mice, melanoma cell line B16 F10, murine T-cell lymphoma RMA-S	Commensal bacteria can stimulate antitumor immune responses via cross-reactivity and bacterial antigens affect the T cell landscape 193.
*Bacteroides fragilis;* *Erysipelotrichaceae*	C57BL/6J and BALB/c mice, mouse colon cancer cell MC38, mouse fibrosarcoma cell line MCA205, mouse colon cancer cell CT26, mouse breast cancer cell 4T1 cell	Ileal microbiota dictates tolerogenic versus immunogenic cell death of ileal intestinal epithelial cells (IECs) and the accumulation of TFH cells in patients 184.
A consortium of 11 bacterial strains	C57BL/6, BALB/c and IQI mice, MC38 adenocarcinoma cell	11 healthy human-associated bacterial strains act together to induce IFN-γ CD8 T cells, confer resistance to the intracellular pathogen *Listeria*, and are effective at inhibiting tumour growth in conjunction with ICIs 194.
*Bacteroides pseudolongum*	C57BL/6J, B6(Cg), Zbtb46tm1(HBEGF)Mnz/J, 129S-Adora2atm1Jfc/J mice, MC38 adenocarcinoma cell, MB49 bladder cancer cell	Intestinal *Bacteroides pseudolongum* activated antitumor T cells through production of the metabolite inosine 212.
*Bacteroides spp.*	C57BL/6 mice, murine colon carcinoma MC38 cell, melanoma cell YUMM1.5	Changes in gut microbiota resulted in enhanced expression of select chemokines and cytokines, which have known roles in the activation of DCs and T cells 199.
*Bifidobacterium*	C57BL/6 and Balb/c mice, murine colon adenocarcinoma cell MC38, murine T cell lymphoma cell EG7.	*Bifidobacterium* potently stimulates STING signaling and increases cross-priming of dendritic cells after anti-CD47 treatment 186.

**Table 2:** Microbiota impact on cancer immunotherapy response.

Immunotherapy	Disease	Microbiota	Mechanism
**Anti-PD-1 treatment**	Melanoma	*Dorea formicigenerans*	The gut bacteria induced maturation of anti-melanoma DCs and T cells, and increased T-cell interferon γ production 263.
	Advanced epithelial tumors	*Akkermansia muciniphilic*	*Akkermansia muciniphila* increased the recruitment of CCR9 +CXCR3 +CD4 + T lymphocytes into mouse tumor beds 168.
	Non-small cell lung cancer	*Enterococcus hirae* strain 13144	*Enterococcus hirae* strain 13144 enhanced peripheral CD8 and CD4 T-cell responses, amplified production of IFN-γ, and an extension of PFS 170.
	Metastatic melanoma, Non-small cell lung cancer, and Renal cell carcinoma	*Akkermansia* and *Prevotella copri*	*Akkermansia* and *Prevotella copri* promoted the recruitment of CCR9+CXCR3+CD4+T lymphocytes to the tumor bed and appeared to be related to type 1 immunity 264,265.
**Anti-PD-L1 treatment**	Melanoma	Live *Bifidobacterium spp.*	Live *Bifidobacterium spp.* inhibited melanoma growth and enhanced tumor-specific CD8 T-cell responses, similar to anti-PD-L1 therapy 168.
**Anti-CTLA4 treatment**	Melanoma	*Burkholderiales* and *Bacteroidales*	The microbiota composition affects IL-12-dependent TH1 immune responses 170.
	Melanoma	*Bacteroides* and theta/thetaaaomicron	*Bacteroides* and theta/thetaaaomicron enhanced LN and intratumoral DC maturation, leading to heightened TH1 immune responses and a restoration of anti-CTLA4 efficacy 169.
**Anti-CTLA4 and anti-PD-1 treatment**	Melanoma	*Holdemania filiformis*, *Bacteroides thetaiotaomicron*, and *Faecalibacterium prausnitzii*	The gut bacteria induced maturation of anti-melanoma DCs and T cells, and increased T-cell interferon γ production 263.

**Table 3:** Clinical trials of microbiota-based therapy modulate the efficacy and AEs of ICl.

NCT number	Cancer types	Interventions	Outcome	Stage
NCT03341143	Melanoma	FMT + Pembrolizumab	Objective Response Rate	Phase 2
NCT03353402	Melanoma	FMT + Anti-PD-1 therapy	Incidence of FMT-related Adverse Events; Proper implant engraftment	Phase 1
NCT03353402	Melanoma	FMT + ICI	Incidence of FMT-related Adverse Events	Phase 1
NCT03686202	Solid Tumors	MET-4 + ICI	Cumulative relative abundance of immunotherapy-responsiveness associated species at day 12 of MET-4; Changes in relative abundance of immunotherapy-responsiveness associated MET-4 strains between baseline and approximately day 12; Number of participants with treatment-related adverse events assessed by CTCAE v.5.0	Phase 2-3
NCT03772899	Melanoma	FMT + Pembrolizumab/Nivolumab	To evaluate the safety of combining FMT using intestinal bacteria existing in the stool of healthy donors with immunotherapy in melanoma patients.	Phase 1
NCT03819296	Solid tumors	FMT + Infliximab/Vedolizumab	Difference in stool microbiome pattern; Incidence of adverse events of FMT	Phase 1-2
NCT03891979	Pancreatic cancer	Pembrolizumab + Ciprofloxacin + Metronidazole	Change in immune activation in pancreatic tumor tissue following treatment with antibiotics and pembrolizumab measured by activation of HLA-DR	Phase 4
NCT04038619	Renal Cell Carcinoma	Loperamide + FMT + ICI	Incidence of FMT-related adverse events; Clinical response/remission of immune-related diarrhea/colitis.	Phase 1
NCT04056026	Mesothelioma	FMT + Keytruda	Progression free survival	Phase 1
NCT04116775	Prostate	FMT + Enzalutamide + Pembrolizumab	Anticancer effect of fecal microbiota transplant from responders to pembrolizumab to non-responders.	Phase 2
NCT04130763	Gastrointestinal	FMT + Anti-PD-1 therapy	Objective Response Rate;Rate of abnormal vital signs and laboratory test results;The number of adverse events	Phase 1
NCT04163289	Renal Cell Carcinoma	FMT + Ipilimumab/Nivolumab	Occurence of immune-related colitis associated with ipilimumab/nivolumab treatment	Phase 1
NCT04163289	Renal Cell Carcinoma	FMT + Nivolumab /lpilimumab	Occurence of immune-related colitis associated with ipilimumab/nivolumab treatment	Phase 1
NCT04264975	Solid Tumors	FMT + ICI	Overall Response Rate	Not Applicable
NCT04521075	Melanoma,Non-small Cell Lung Cancer	FMT + Nivolumab	Incidence of FMT-related Adverse Events; Overall Response Rate	Phase 1-2
NCT04577729	Melanoma	FMT + ICI	Progression free survival	Not Applicable
NCT04699721	Non-small Cell Lung Cancer	*Bifidobacterium trifidum* live powder + Nivolumab + Paclitaxel + Carboplatin AUC5	Adverse effects;Surgical complications; Non-R0 surgical events	Phase 1
NCT04729322	Colorectal cancer	FMT + Pembrolizumab/Nivolumab	Objective response rate	Phase 1
NCT04758507	Renal Cell Carcinoma	FMT + ICI	Number of participants who will be free from tumor progression, as assessed by RECIST criteria v. 1.1.	Phase 1-2
NCT04883762	Solid tumors	FMT + ICI	Incidence of FMT-related adverse events	Phase 1
NCT04924374	Advanced Lung Cancer	FMT + Anti-PD-1 therapy	Measure of safety	Not Applicable
NCT04988841	Melanoma	FMT + Pembrolizumab /Nivolumab	To assess whether the safety of a 23-week treatment with MaaT013, combined with ipilimumab+nivolumab, is different from that of ipilimumab+nivolumab+placebo in patients with melanoma naïve to Ipilimumab and anti-PD-1	Phase 2
NCT05008861	Non-Small Cell Lung Cancer	FMT+Anti-PD-1/PD-L1 therapy+Platinum based chemotherapy	Incidence of FMT-related Adverse Events;Incidence of anti-PD-1/PD-L1-related Adverse Events	Phase 1
NCT05220124	Bladder Urothelial Carcinoma	*Bifidobacterium Lactobacillus* and *Enterococcus* Capsules+ICI	Progression-free survival	Phase 4
NCT05251389	Melanoma Stage IIIMelanoma Stage IV	FMT+ICI	Efficacy, defined as clinical benefit (SD, PR, CR)	Phase 1Phase 2
NCT05279677	Colorectal Cancer	FMT + Sintilimab + Fruquintinib	Overall Response Rate	Phase 2
NCT05286294	Melanoma; Head and Neck Squamous Cell Carcinoma; Cutaneous Squamous Cell Carcinoma; MSI-High; Clear Cell Renal Cell Carcinoma; Non-small Cell Lung Cancer	FMT+ICI	Safety evaluation of FMT in advanced cancer patients;Tumor response evaluation	Phase 2
NCT05462496	Pancreatic cancer	FOLFIRINOX + Ciprofloxacin + Metronidazole + Pembrolizumab	Achievement of overall immune response	Phase 2
NCT05690048	Hepatocellular carcinoma	FMT/Vancomycin + Atezolizumab + Bevacizumab	Differential tumoral CD8 T-cell infiltration; Adverse event documentation of FMT in advanced HCC	Phase 2
NCT05750030	Hepatocellular carcinoma	FMT + Atezolizumab + Bevacizumab	Safety of atezolizumab/bevacizumab in combination with FMT, measured by incidence and severity of treatment-related adverse events, determined according to National Cancer Institute CTCAE v.5.0.	Phase 2

Abbreviations: FMT: Fecal microbiota transplantation; MET: Microbial ecosystem therapeutics; SD: stable disease, PR: partial response, CR: complete response; PD-1: programmed cell death protein 1; PD-L1: Programmed cell death 1 ligand 1; ICI: Immune checkpoint inhibitor.

**Table 4:** The Impact of antibiotics on cancer immunotherapy efficacy.

Disease	Therapy	Antibiotic intervention	Outcome
Advancer non-small cell lung cancer, renal cell carcinoma and urothelial carcinoma	Anti-PD-1 and anti-PD-L1 treatment	Retrospective analysis of the antibiotic utilization of patients within a window of 60 days before and 30 days after the start of treatment ICI initiation.	Patients who have undergone antibiotic treatment experience a poorer prognosis 170.
Metastatic renal cell carcinoma	Systemic therapy	Oral or intravenous systemic antibiotic treatment	Antibiotic use was associated with worse outcomes in patients treated with either contemporary PD-1/PD-L1-based ICIs or cytokines 284.
Metastatic renal cell carcinoma	Anti-CTLA4 and anti-PD-1 treatment	Retrospective analysis of the antibiotic utilization of patients within a window of 30 days before and 30 days after the start of treatment ICI initiation.	Use of antibiotic before ICIs treatment is a predictor of poor ICIs response in metastatic renal cell carcinoma 285.
Non-small cell lung cancer	Anti-PD-L1 treatment	Retrospective analysis of the antibiotic utilization of patients in the 1 month prior to ICI initiation.	Antibiotics use in patients with metastatic non-small cell lung cancer is associated with poor outcome and may influence the efficacy of ICI 286.
Multiple advanced cancers	Anti-PD-1 and anti-PD-L1 treatment	Retrospective analysis of the antibiotic utilization of patients within a window of 14 days before and 14 days after the start of treatment ICI initiation.	Patients who have undergone antibiotic treatment experience a poorer prognosis 288.
Advanced non-small cell lung cancer	Anti-PD-1 and anti-PD-L1 treatment	Retrospective analysis the antibiotic utilization of patients in the 1 month prior to ICI initiation.	Patients who have undergone antibiotic treatment exhibit lower alpha diversity in the gut microbiota and experience a poorer prognosis 290.
Renal cell carcinoma and non-small-cell lung cancer	Anti-PD-1, anti-PD-L1 and anti-CTLA4 treatment	Retrospective analysis of the antibiotic utilization of patients in the 2 months prior to ICI initiation.	Patients who have received antibiotic treatment exhibit a poorer prognosis, and those who received antibiotics within the 30 days preceding ICIs therapy have a worse prognosis compared to patients who received antibiotics within the 60 days preceding ICI therapy^291^.
Renal Cell Carcinoma	Anti-PD-1 treatment	Retrospective analysis of the antibiotic utilization of patients in the 2 months prior to ICI initiation.	Patients who have undergone antibiotic treatment experience a poorer prognosis 175.
Multiple cancers	ICIs	Prospective analysis of the antibiotic utilization of patients up to 30 days prior to or concurrent with ICI therapy.	The administration of antibiotics up to 30 days prior to ICIs therapy is associated with diminished efficacy of ICI treatment, and this phenomenon is observed independent of the specific tumor site 299.

## Abbreviations

ACT: Adoptive cell therapy
APCs: Antigen-presenting cells
CAR-T: Chimeric antigen receptor T-cell
CCL5, better known as RANTES: Chemokine (C-C motif) ligand 5
CD: Cluster of differentiation
CEACAM1: Carcinoembryonic antigen cell adhesion molecule 1
CIN2/3: cervical intraepithelial neoplasia 2/3
c-Myc: Cellular myelocytomatosis
CRC: Colorectal cancer
CTL: Cytotoxic T lymphocyte
CTLA4: Cytotoxic T-lymphocyte-associated antigen 4
CXCR4: C-X-C chemokine receptor type 4
DC: Dendritic cell
DNA: Deoxyribonucleic acid
DOXO: Doxorubicin
EBV: Epstein-Barr virus
ERK: Extracellular regulated protein kinases
FDA: Food and drug administration
FMT: Fecal microbiota transplantation
FN-I: Fibronectin I
GALT: gut-associated lymphoid tissues
GF: Germ-free
GITR: glucocorticoid-induced TNF receptor
GM-CSF: Granulocyte-macrophage colony-stimulating factor
Gpr109a: G-coupled receptor-109a
GVAX: GM-CSF gene transduced autologous tumor vaccine
GVHD: Graft-versus-host disease
HBV: Hepatitis B virus
HCC: Hepatocellular carcinoma
HER2: Human epidermal growth factor receptor 2
HLA: Human leukocyte antigen
HSCT: Hematopoietic stem cell transplantation
HSV-1: Herpes simplex virus type 1
ICAM-1: Intercellular adhesion molecule-1
ICI: Immune checkpoint inhibitor
ICOS: Inducible T-cell costimulator
IFN: Interferon
IGRP: Islet-specific glucose-6-phosphatase catalytic subunit-related protein
IL-2: Interleukin-2
iPSC: induced pluripotent stem cell
irAEs: Immune-related adverse events
ITAM: Immunoreceptor tyrosine-based activation motifs
JNK: Jun N-terminal kinase
LAG3: Lymphocyte-activation gene 3
LN: lymph node
LPS: lipopolysaccharide
MAGE: Melanoma-associated antigen
MAPK: Mitogen-activated protein kinase
MCPyV: Merkel cell polyomavirus
MDSC: Myeloid-derived suppressor cells
MEK: Mitogen-activated extracellular signal-regulated kinase
MeV: Measles virus
MHC: Major histocompatibility complex
NF-κB: Nuclear factor κappa-B
NK: Natural killer
NOX2: Nicotinamide adenine dinucleotide phosphate oxidase 2
NSCLC: Non-small cell lung cancer
OMV: Outer membrane vesicles
ORR: Objective response rate
OS: Overall survival
PBL: Peripheral blood lymphocyte
PD-1: Programmed cell death-1
PDAC: pancreatic ductal adenocarcinoma
PD-L1: Programmed cell death ligand-1
PFS: Progression-free survival
PMP: Precursor myeloproliferation
PSMB4: Proteasome 20S subunit beta 4
PTX: Paclitaxel
PVR: Poliovirus receptor
RCC: Renal cell carcinoma
RET: Rarranged during transfection
RIG-I: Retinoic acid-inducible gene I
RNA: Ribonucleic acid
RNF5: Ring finger protein 5
ROS: Reactive Oxygen Species
SCFAs: Short-chain fatty acids
scFv: Single-chain variable fragments
SPF: Specific pathogen-free
STING: Stimulator of interferon genes
TAM: Tumor-associated macrophage
TBI: total body irradiation
TCR: T cell receptor
TFH: Follicular helper T
TIGIT: T-cell immunoglobulin and ITIM domain
TILs: Tumor-infiltrating lymphocytes
TIM3: T-cell immunoglobulin and mucin domain-containing protein 3
TLR4: Toll-like receptor 4
TME: Tumor microenvironment
TNF: tumor necrosis factor
Tregs: Regulatory T cells
T-VEC: Talimogene laherparepvec
ZAP2: Zeta-chain-associated protein kinase**.**
